# Identification of UDP-rhamnosyltransferases and UDP-galactosyltransferase involved in flavonol glycosylation in *Morella rubra*

**DOI:** 10.1093/hr/uhac138

**Published:** 2022-06-20

**Authors:** Chuanhong Ren, Yan Guo, Linfeng Xie, Zhikang Zhao, Mengyun Xing, Yunlin Cao, Yilong Liu, Jing Lin, Donald Grierson, Bo Zhang, Changjie Xu, Kunsong Chen, Xian Li

**Affiliations:** Zhejiang Provincial Key Laboratory of Horticultural Plant Integrative Biology, Zhejiang University, Zijingang Campus, Hangzhou 310058, China; The State Agriculture Ministry Laboratory of Horticultural Plant Growth, Development and Quality Improvement, Zhejiang University, Zijingang Campus, Hangzhou 310058, China; Zhejiang Provincial Key Laboratory of Horticultural Plant Integrative Biology, Zhejiang University, Zijingang Campus, Hangzhou 310058, China; The State Agriculture Ministry Laboratory of Horticultural Plant Growth, Development and Quality Improvement, Zhejiang University, Zijingang Campus, Hangzhou 310058, China; Zhejiang Provincial Key Laboratory of Horticultural Plant Integrative Biology, Zhejiang University, Zijingang Campus, Hangzhou 310058, China; The State Agriculture Ministry Laboratory of Horticultural Plant Growth, Development and Quality Improvement, Zhejiang University, Zijingang Campus, Hangzhou 310058, China; Zhejiang Provincial Key Laboratory of Horticultural Plant Integrative Biology, Zhejiang University, Zijingang Campus, Hangzhou 310058, China; The State Agriculture Ministry Laboratory of Horticultural Plant Growth, Development and Quality Improvement, Zhejiang University, Zijingang Campus, Hangzhou 310058, China; Zhejiang Provincial Key Laboratory of Horticultural Plant Integrative Biology, Zhejiang University, Zijingang Campus, Hangzhou 310058, China; The State Agriculture Ministry Laboratory of Horticultural Plant Growth, Development and Quality Improvement, Zhejiang University, Zijingang Campus, Hangzhou 310058, China; Zhejiang Provincial Key Laboratory of Horticultural Plant Integrative Biology, Zhejiang University, Zijingang Campus, Hangzhou 310058, China; The State Agriculture Ministry Laboratory of Horticultural Plant Growth, Development and Quality Improvement, Zhejiang University, Zijingang Campus, Hangzhou 310058, China; Zhejiang Provincial Key Laboratory of Horticultural Plant Integrative Biology, Zhejiang University, Zijingang Campus, Hangzhou 310058, China; The State Agriculture Ministry Laboratory of Horticultural Plant Growth, Development and Quality Improvement, Zhejiang University, Zijingang Campus, Hangzhou 310058, China; Zhejiang Provincial Key Laboratory of Horticultural Plant Integrative Biology, Zhejiang University, Zijingang Campus, Hangzhou 310058, China; The State Agriculture Ministry Laboratory of Horticultural Plant Growth, Development and Quality Improvement, Zhejiang University, Zijingang Campus, Hangzhou 310058, China; Zhejiang Provincial Key Laboratory of Horticultural Plant Integrative Biology, Zhejiang University, Zijingang Campus, Hangzhou 310058, China; The State Agriculture Ministry Laboratory of Horticultural Plant Growth, Development and Quality Improvement, Zhejiang University, Zijingang Campus, Hangzhou 310058, China; Plant and Crop Sciences Division, School of Biosciences, University of Nottingham, Sutton Bonington Campus, Loughborough, LE12 5RD, UK; Zhejiang Provincial Key Laboratory of Horticultural Plant Integrative Biology, Zhejiang University, Zijingang Campus, Hangzhou 310058, China; The State Agriculture Ministry Laboratory of Horticultural Plant Growth, Development and Quality Improvement, Zhejiang University, Zijingang Campus, Hangzhou 310058, China; Zhejiang Provincial Key Laboratory of Horticultural Plant Integrative Biology, Zhejiang University, Zijingang Campus, Hangzhou 310058, China; The State Agriculture Ministry Laboratory of Horticultural Plant Growth, Development and Quality Improvement, Zhejiang University, Zijingang Campus, Hangzhou 310058, China; Zhejiang Provincial Key Laboratory of Horticultural Plant Integrative Biology, Zhejiang University, Zijingang Campus, Hangzhou 310058, China; The State Agriculture Ministry Laboratory of Horticultural Plant Growth, Development and Quality Improvement, Zhejiang University, Zijingang Campus, Hangzhou 310058, China; Zhejiang Provincial Key Laboratory of Horticultural Plant Integrative Biology, Zhejiang University, Zijingang Campus, Hangzhou 310058, China; The State Agriculture Ministry Laboratory of Horticultural Plant Growth, Development and Quality Improvement, Zhejiang University, Zijingang Campus, Hangzhou 310058, China

## Abstract

Flavonol glycosides are health-promoting phytochemicals important for human nutrition and plant defense against environmental stresses. Glycosylation modification greatly enriches the diversity of flavonols. *Morella rubra*, a member of the Myricaceae, contains high amounts of myricetin 3-*O*-rhamnoside (M3Rha), quercetin 3-*O*-rhamnoside (Q3Rha), and quercetin 3-*O*-galactoside (Q3Gal). In the present study, MrUGT78R1 and MrUGT78R2 were identified as two functional UDP-rhamnosyltransferases, while MrUGT78W1 was identified as a UDP-galactosyltransferase. Site-directed mutagenesis identified Pro143 and Asn386 as important residues for rhamnosyl transfer activity of MrUGT78R1, while the two corresponding positions in MrUGT78W1 (i.e. Ser147 and Asn370) also play important roles in galactosyl transfer activity. Transient expression data for these three MrUGTs in *Nicotiana benthamiana* tested the function of MrUGT78R1 and MrUGT78R2 as rhamnosyltransferases and MrUGT78W1 as a galactosyltransferase in glycosylation of flavonols. This work enriches knowledge of the diversity of UDP-rhamnosyltransferase *in planta* and identifies two amino acid positions important for both rhamnosyltransferase and galactosyltransferase.

## Introduction

Flavonol glycosides are bioactive flavonoids important for human health [1–3]. In plants, they are important for development and the defense system [4–6]. Thousands of flavonol glycosides have been discovered so far, although the number of flavonol aglycones is quite limited. The most common flavonol aglycones include kaempferol (K), quercetin (Q), and myricetin (M). Glycosylation is one of the modifications that enriches the diversity of flavonols [7]. Glycosylation improves the stability, solubility, and transferability of flavonols [8–10].

UDP-glycosyltransferases (UGTs) are the largest group of plant glycosyltransferases (GTs, EC 2.4.x.y,) according to the CAZy database (http://www.cazy.org). They catalyze glycosylation of flavonols, which occurs during the later stages of flavonol biosynthesis. The most commonly used sugar donors for UGTs include UDP-glucose (UDP-Glc), UDP-galactose (UDP-Gal), and UDP-rhamnose (UDP-Rha) [7, 11]. So far, many glucosyltransferases, such as AtUGT78D2 [12], FaGT6, FaGT7 [13], CsUGT78A14 [14], and MdUGT71B1 [11], and several galactosyltransferases, such as F3GalTase [15], CsUGT78A15 [14], and MdUGT75B1 [11], have been identified as UGTs involved in flavonol glycosylation. However, few rhamnosyltransferases have been reported compared with glucosyltransferases and galactosyltransferases.

*Morella rubra* (Chinese bayberry), a member of the Myricaceae, is a rich source of flavonol glycosides and abundant myricetin 3-*O*-rhamnoside (M3Rha, myricitrin), quercetin 3-*O*-rhamnoside (Q3Rha), and quercetin 3-*O*-galactoside (Q3Gal) have been reported in the fruit [16]. *M. rubra* has significant medical values in Chinese folk medicine and various flavonol glycosides, such as M3Rha, play important roles in bioactivities such as antioxidant [17, 18], antidiabetic [19–21], anti-inflammatory [22], and anti-atherosclerosis [23] agents. Recently, a high content of M3Rha was detected in *M. rubra* [>10 mg g^−1^ fresh weight (FW) in leaves or flowers] [24]. Therefore, *M. rubra* is an ideal plant material for unraveling the rhamnosyltransferases of flavonols.

Rapid developments of genomics and bioinformatics have greatly facilitated the discovery and identification of functionally diverse UGTs from this vast gene family [25–27]. Usually, >100 UGT members can be found in a single plant species [27]. In *Arabidopsis*, *AtUGT78D1* and *AtUGT78D2* encode two flavonol-specific glycosyltransferases, and their mutants *ugt78d1* and *ugt78d2* contain much smaller total flavonol contents and quite different flavonol glycoside compositions [4, 28]. The plant secondary product glycosyltransferase (PSPG) box is a conserved motif close to the C-terminal of plant UGTs, and it is critical for sugar donor preference [25, 29]. For example, UDP-glucosyltransferases usually have glutamine (Gln) as the last amino acid residue of the PSPG box, and examples of such UGTs include AtUGT78D2 [12] and CsUGT78A14 from tea (*Camellia sinensis*) [14] and FaGT6 and FaGT7 from strawberry [13]. However, such sugar donor preferences are not solely determined by one single amino acid in the PSPG box and more functional studies should be carried out with plant resources that accumulate different flavonol glycosides in order to investigate the determinants of UGT sugar donor specificity.

Recently, both the transcriptome and genome of *M. rubra* have been reported [30, 31], and this has laid the foundation for the functional characterization of UGTs involved in the glycosylation of flavonols in this plant. Here, MrUGT78R1 and MrUGT78R2 were identified as flavonol-specific UDP-rhamnosyltransferases and MrUGT78W1 was identified as a UDP-galactosyltransferase by enzymatic analysis and transient overexpression in *Nicotiana benthamiana*. By molecular docking and site-directed mutagenesis analysis, Pro143 and Asn386 of MrUGT78R1 were identified as important residues for its rhamnosyl transfer activity, while Ser147 and Asn370 of MrUGT78W1 were identified as important residues for its galactosyl transfer activity.

## Results

### Flavonol glycoside accumulation in *M. rubra*

**Figure 1:**
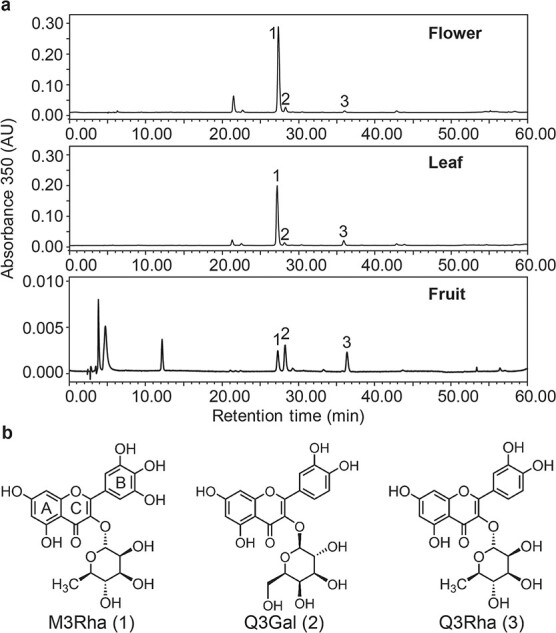
Different flavonol glycosides accumulate in *M. rubra*. **a** HPLC chromatograms of main flavonol glycosides detected in flower, leaf, and mature fruit of ‘Biqi’ cultivar. Peak 1, myricetin 3-*O*-rhamnoside (M3Rha); peak 2, quercetin 3-*O*-galactoside (Q3Gal); peak 3, quercetin 3-*O*- rhamnoside (Q3Rha). **b** Structures of main flavonol glycosides in *M. rubra*. A, B, and C rings of the flavonoid skeleton are labeled.

**Figure 2:**
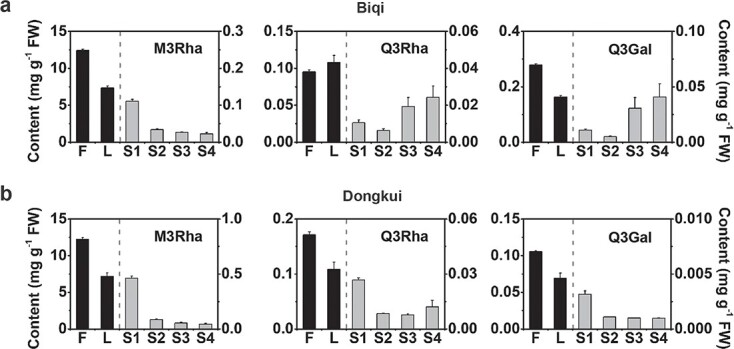
Accumulation of flavonol glycosides in different tissues of *M. rubra*. **a** Accumulation patterns of three main flavonol glycosides, myricetin 3-*O*-rhamnoside (M3Rha), quercetin 3-*O*-rhamnoside (Q3Rha), and quercetin 3-*O*-galactoside (Q3Gal) in flowers (F), leaves (L), and fruit of different development stages (S1–S4) in ‘Biqi’ cultivar. **b** M3Rha, Q3Rha, and Q3Gal accumulation pattern in ‘Dongkui’ cultivar. The *y*-axis on the left represents contents in flowers and leaves (black columns). The *y*-axis on the right represents contents in fruit (gray columns). Data are mean ± standard error (*n* = 3).

Flavonol glycosides for different tissues of ‘Biqi’ and ‘Dongkui’ cultivars were analyzed by HPLC. Three main flavonol glycosides, i.e. M3Rha, Q3Rha, and Q3Gal, were identified and quantified (Fig. 1), which was consistent with our previous study [24]. M3Rha was the main flavonol glycoside that accumulated in flowers, leaves, and young fruits of both cultivars, and it reached 12.42 ± 0.16 and 12.22 ± 0.22 mg g^−1^ FW in flowers of ‘Biqi’ (Fig. 2a) and ‘Dongkui’ (Fig. 2b), respectively. Younger fruits (S1) of both cultivars accumulated higher levels of M3Rha than mature fruits in terms of FW. Q3Rha and Q3Gal accumulated in flowers and leaves of both cultivars, and both significantly accumulated during fruit development in ‘Biqi’ (Fig. 2a) but not in ‘Dongkui’ (Fig. 2b).

### Isolation of MrUGT78R1, MrUGT78R2, and MrUGT78W1

**Figure 3:**
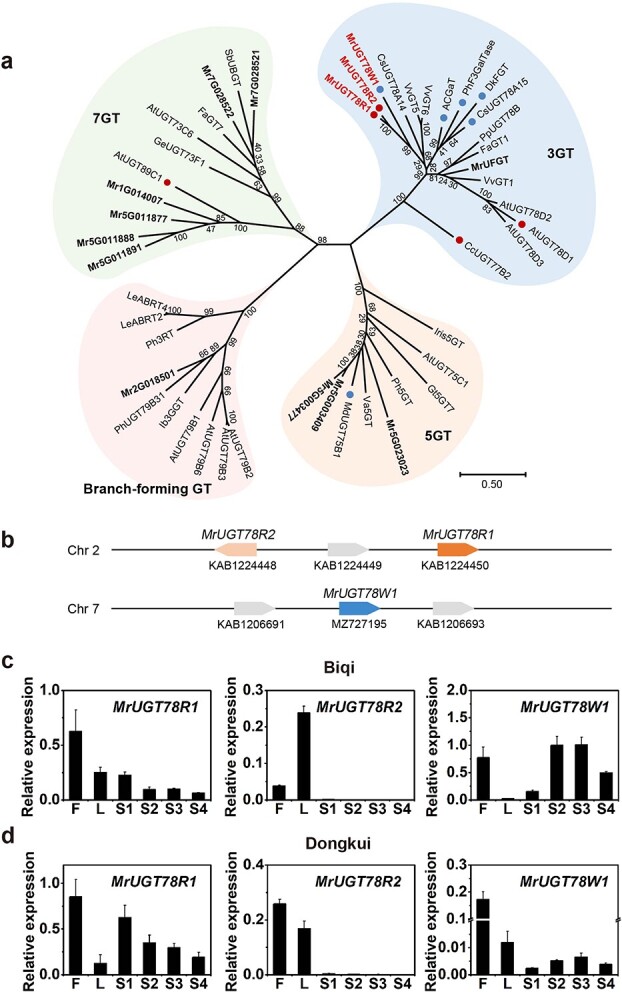
Phylogenetic and gene expression analysis of MrUGTs. **a** Phylogenetic analysis of MrUGTs and UGTs from other species performed by the maximum likelihood method using MEGA-X. Bootstrap values shown on branches are from 1000 replicates. Bar represents 0.5 amino acid substitutions per site. MrUGTs are marked in bold. Red dots represent rhamnosyltransferases and blue dots represent galactosyltransferases. MrUGTs characterized in this work are marked in red font. The abbreviations of species names are follows: At, *Arabidopsis*; AC, *Aralia cordata*; Cs, *Camellia sinensis*; Cc, *Crocosmia × crocosmiiflora*; Dk, *Diospyros kaki*; Fa, *Fragaria × ananassa*; Gt, *Gentiana triflora*; Ge, *Glycyrrhiza echinata*; Ib, *Ipomoea batatas*; Iris, *Iris hollandica*; Le, *Lobelia erinus*; Md, *Malus × domestica*; Ph, *Petunia hybrida*; Pp, *Prunus persica*; Sb, *Scutellaria baicalensis*; Va, *Vitis amurensis*; Vv, *Vitis vinifera*; Mr, *Morella rubra*. Accession numbers of known UGTs in the phylogenetic tree are shown in Supplementary Table S1. **b** Graphical map of *MrUGT*s in the *M. rubra* genome. GenBank accession numbers are shown below each gene. **c** Expression pattern of *MrUGT78R1*, *MrUGT78R2*, and *MrUGT78W1* in flowers (F), leaves (L), and fruit development stages (S1–S4) of the ‘Biqi’ cultivar. **d** Expression pattern of *MrUGT78R1*, *MrUGT78R2*, and *MrUGT78W1* in the ‘Dongkui’ cultivar. Data are mean ± standard error (*n* = 3).

Flavonol glycosylation mostly occurs on the 3-OH of the C ring of the flavonol aglycones in *M. rubra* (Fig. 1). Since most known plant 3-*O*-UDP-glycosyltransferases belong to the 3GT subcluster of the UGT family [12], we selected our candidate genes based on phylogenetic analysis of the 3GT members from *M. rubra*. Among the UGT sequences in the *M. rubra* genome, only four MrUGTs belong to the 3GT cluster, i.e. MrUGT78R1, MrUGT78R2, MrUGT78W1, and MrUFGT (Fig. 3a). MrUFGT was reported as a UGT involved in anthocyanin biosynthesis in *M. rubra* [32]. Therefore, we chose *MrUGT78R1*, *MrUGT78R2*, and *MrUGT78W1* as our candidate genes for flavonol glycosylation. Several other MrUGTs that clustered in 5GT, 7GT, and branch-forming GT clusters are also shown in Fig. 3a.

*MrUGT78R1*, *MrUGT78R2*, and *MrUGT78W1* were cloned from cDNA libraries of both ‘Biqi’ and ‘Dongkui’ and no differences in coding sequences were found between the genes from the two cultivars. Open reading frames (ORFs) of *MrUGT78R1*, *MrUGT78R2*, and *MrUGT78W1* were 1455, 1443, and 1410 bp, which encoded proteins containing 484, 480, and 469 amino acid residues, respectively. Amino acid sequences of MrUGT78R1 and MrUGT78R2 shared 85.33% identity. MrUGT78R1 and MrUGT78W1 shared 52.86% identity, and MrUGT78R2 shared 52.06% identity with MrUGT78W1. Phylogenetic analysis showed that these three MrUGTs showed the highest homology with CsUGT78A14 (49.69, 49.69, and 47.25%, respectively) from *C. sinensis*. Multiple sequence alignment analysis of MrUGT78R1, MrUGT78R2, and MrUGT78W1 found that the PSPG box close to the C-terminal end was conserved (Supplementary Data Fig. S1). The location of these three MrUGTs in the genome of the *M. rubra* was also investigated. *MrUGT78R1* and *MrUGT78R2* were located in the reverse orientation on Chromosome 2 (Fig. 3b), while *MrUGT78W1* was located on Chromosome 7 (Fig. 3b).

### Transcript profiles of *MrUGT78R1*, *MrUGT78R2*, and MrUGT78W1 in *M. rubra*

Transcript abundances of *MrUGT78R1*, *MrUGT78R2*, and *MrUGT78W1* were investigated by quantitative real-time–PCR (qRT–PCR) in different organs of both cultivars. Gene expression levels of all three *MrUGTs* were highest in flowers, except for *MrUGT78R2* in ‘Biqi’ (Fig. 3c and d). *MrUGT78R1* was more highly expressed than *MrUGT78R2* in all tissues tested, and gene expression of *MrUGT78R2* was at a very low level in fruit of both cultivars (Fig. 3c and d). During fruit development, gene expression of *MrUGT78R1* was the highest at S1 stage in both cultivars (Fig. 3c and d), which was in agreement with the high accumulation of M3Rha at S1 stage in both cultivars (Fig. 2a and b). Expression of *MrUGT78W1* was upregulated significantly during development of ‘Biqi’ fruit (Fig. 3c), while expression of *MrUGT78W1* remained at a low level throughout the development of ‘Dongkui’ fruit (Fig. 3d).

### Enzymatic assays of recombinant MrUGT78R1, MrUGT78R2, and MrUGT78W1

**Figure 4:**
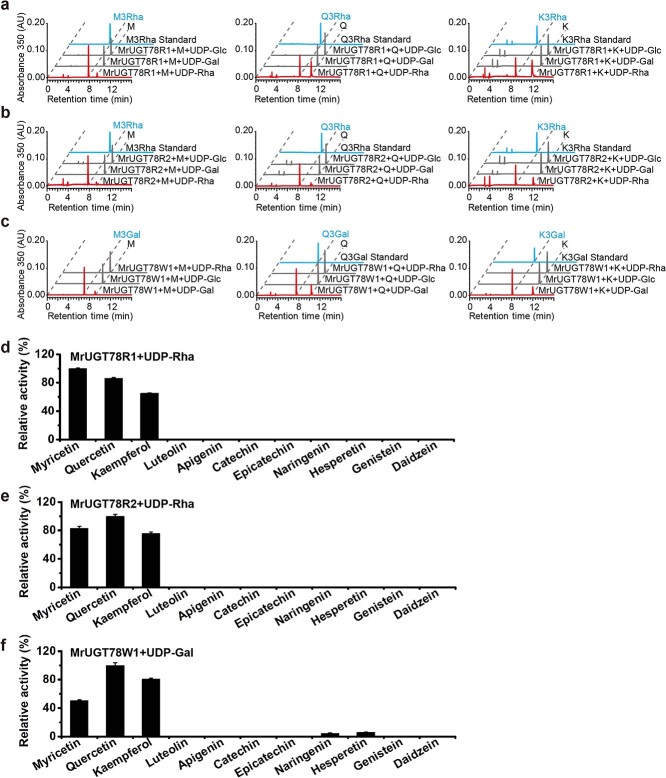
Substrate specificity analysis of recombinant MrUGTs. **a** Enzyme activity analysis of recombinant MrUGT78R1 with UDP-rhamnoside (UDP-Rha, red line), UDP-galactoside (UDP-Gal, gray line), and UDP-glucoside (UDP-Glc, gray line) as sugar donors. **b** Enzyme activity analysis of recombinant MrUGT78R2 with UDP-Rha (red line), UDP-Gal (gray line), and UDP-Glc (gray line) as sugar donors. **c** Enzyme activity analysis of recombinant MrUGT78W1 with UDP-Gal (red line), UDP-Glc (gray line), and UDP-Rha (gray line) as sugar donors. Flavonol aglycones myricetin (M), quercetin (Q) and kaempferol (K) were used as sugar acceptors for the three MrUGTs. **d**–**f** Relative enzyme activities of recombinant MrUGT78R1 with UDP-Rha (**d**), MrUGT78R2 with UDP-Rha (**e**), and MrUGT78W1 with UDP-Gal (**f**) toward various flavonoids. Data are mean ± standard error (*n* = 3).

Recombinant MrUGT proteins produced by *Escherichia coli* were analyzed by SDS–PAGE (Supplementary DataFig. S2). Recombinant protein activity was evaluated with sugar donors UDP-Rha, UDP-Gal, and UDP-Glc, and sugar acceptors M, Q, and K. Results indicated that both MrUGT78R1 and MrUGT78R2 proteins could only transfer UDP-Rha to flavonol aglycones. By comparing the retention time and fragmentation pattern with standards, product peaks with *m*/*z* 463, 447, and 431 were identified as M3Rha, Q3Rha, and kaempferol 3-*O*-rhamnoside (K3Rha), respectively (Fig. 4a and b, Supplementary Data Fig. S3). MrUGT78R1 and MrUGT78R2 did not show activity towards UDP-Glc or UDP-Gal for M, Q, and K (Fig. 4a and b). MrUGT78W1 protein could only transfer UDP-Gal into flavonol aglycones. Based on fragmentation information, product peaks with *m*/*z* 479 were tentatively identified as myricetin 3-*O*-galactoside (M3Gal) (Fig. 4c, Supplementary Data Fig. S3). By comparing the retention time and fragmentation information with standards, product peaks with *m*/*z* 463, and 447 were identified as Q3Gal and kaempferol 3-*O*-galactoside (K3Gal) respectively (Fig. 4c, Supplementary Data Fig. S3). MrUGT78W1 did not show activity towards UDP-Rha or UDP-Glc for M, Q, or K (Fig. 4c). Using UDP-Rha as sugar donor, the optimum reaction conditions for MrUGT78R1 were pH 6.0 and 50°C (Supplementary Data Fig. S4a), while the optimum reaction conditions for MrUGT78R2 were pH 7.5 and 35°C (Supplementary Data Fig. S4b). Using UDP-Gal as sugar donor, the optimum reaction conditions for MrUGT78W1 were pH 8.5 and 30°C (Supplementary Data Fig. S4c).

The enzyme activities of three MrUGTs towards different flavonoid aglycones were also tested. MrUGT78R1 and MrUGT78R2 exhibited strict regiospecific activity towards flavonol aglycones (M, Q, K), and had no rhamnosyl transfer activity towards luteolin, apigenin, catechin, epicatechin, naringenin, hesperetin, genistein, or daidzein (Fig. 4d and e). MrUGT78W1 displayed strong activities towards flavonol aglycones (M, Q, K), while it showed weak activity towards flavanones naringenin and hesperetin (Fig. 4f), indicating that the enzyme can also glycosylate other flavonoids.

Enzymatic kinetic properties of three MrUGTs were determined in the linear range of flavonol aglycone (M, Q, K) concentrations (Supplementary Data Fig. S5). Using UDP-Rha as sugar donor, both MrUGT78R1 and MrUGT78R2 showed small differences in *K*_m_ towards the three flavonol aglycones tested (Table 1, Supplementary Data Fig. S5). However, MrUGT78R1 showed the highest *K*_cat_ towards M while MrUGT78R2 showed the highest *K*_cat_ towards Q (Table 1), which is consistent with the results in Fig. 4d and e. Using UDP-Gal as sugar donor, MrUGT78W1 had the highest *K*_cat_ towards Q (Table 1), which is consistent with the results in Fig. 4f.

### Site-directed mutagenesis of MrUGT78R1 and MrUGT78W1

**Figure 5:**
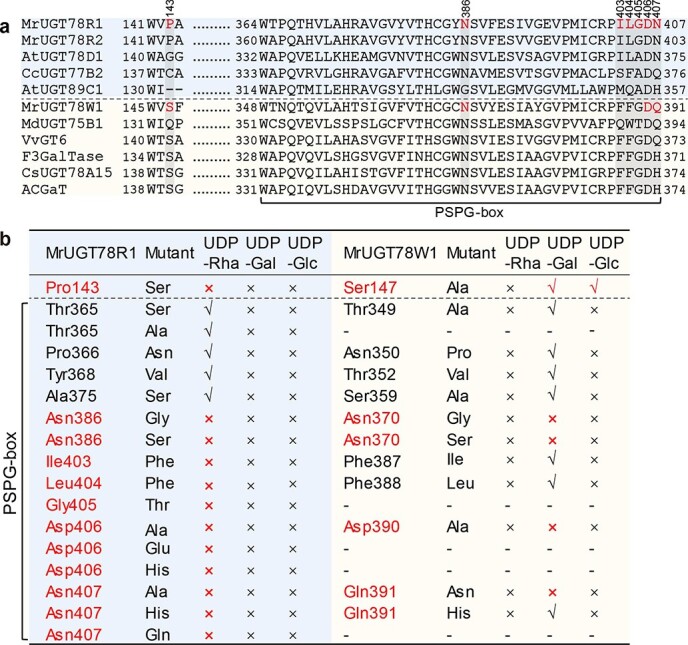
Analysis of enzymatic activity upon site-directed mutagenesis of MrUGT78R1 and MrUGT78W1. **a** Multiple sequence alignments of UGTs. UDP-rhamnosyltransferases are highlighted with a light blue background above the dashed line. UDP-galactosyltransferases are shown with a light yellow background below the dashed line. Important amino acid residues for UDP-sugar transfer activity of MrUGTs are marked in red. Numbers above the alignment indicate the amino acid position in MrUGT78R1. Numbering for each protein represents the first and last residues. Amino acid residues in the PSPG box are indicated below. **b** Site-directed mutagenesis analysis of MrUGT78R1 (light blue background on left) and MrUGT78W1 (light yellow background on right) with Q and different UDP-sugar donors as substrates. Amino acid residues important for UDP-sugar transfer activity of MrUGTs are marked in red. Results with specific amino acid substitutions in MrUGT78R1 and MrUGT78W1 are shown in separate rows.

To explore the key amino acids underlying UDP-Rha- and UDP-Gal-specific catalytic activity of plant UGTs, molecular docking analysis of UDP-sugar-enzymes and amino acid sequence alignment were carried out (Fig. 5a, Supplementary Data Fig. S6a and b). Amino acids in the binding pocket of MrUGT78R1 and MrUGT78W1 were selected for site-directed mutagenesis (Supplementary Data Fig. S6c and d). For MrUGT78R1, all mutated amino acids except for Pro143 were part of the PSPG box. Results showed that in MrUGT78R1, mutations of Pro143 to Ser, Asn386 to Gly or Ser, Ile403 to Phe, Leu404 to Phe, Gly405 to Thr, Asp406 to Ala or Glu, or His, and Asn407 to Ala or His or Gln resulted in complete loss of rhamnosyl transfer activity (Fig. 5b). Proteins with replacement of Thr365 with Ser or Ala, Pro366 with Asn, Tyr368 with Val, and Ala375 with Ser retained rhamnosyl transfer activity (Fig. 5b). No mutations of MrUGT78R1 resulted in any additional galactosyl transfer or glucosyl transfer activity (Fig. 5b, Supplementary Data Fig. S7).

In MrUGT78W1, all mutated amino acids except for Ser147 were part of the PSPG box. Mutations replacing Asn370 with Gly or Ser, Asp390 with Ala, and the last amino acid of the PSPG box Gln391 with Asn resulted in complete loss of galactosyl transfer activity (Fig. 5b, Supplementary Data Fig. S8). Replacement of Thr349 with Ala, Asn350 with Pro, Thr352 with Val, Ser359 with Ala, Phe387 with Ile, Phe388 with Leu, and Gln391 with His retained galactosyl transfer activity (Fig. 5b, Supplementary Data Fig. S8). Mutation of Ser147 to Ala generated a protein that still retained galactosyl transfer activity and in addition acquired glucosyl transfer activity but no rhamnosyl transfer activity (Fig. 5b, Supplementary Data Fig. S8). No other MrUGT78W1 mutation produced additional rhamnosyl transfer or glucosyl transfer activity besides Ser147 (Fig. 5b, Supplementary Data Fig. S8).

**Table 1:** Kinetic parameters of recombinant MrUGT78R1, MrUGT78R2, and MrUGT78W1. Data are mean ± standard error, with expressed purified proteins prepared in three separate batches.

**Enzyme**	**Substrate**	***K***_**m**_ **(μM)**	***K***_**cat**_ **(10** ^**3**^ **S**^**−1**^**)**	***K***_**cat**_ **/*K***_**m**_ **(10** ^**4**^ **S**^**−1**^ **M**^**−1**^**)**
MrUGT78R1	Myricetin	68.11 ± 2.74	1.04 ± 0.04	1.52 ± 0.01
	Quercetin	36.05 ± 0.63	0.50 ± 0.03	1.39 ± 0.06
	Kaempferol	23.64 ± 2.26	0.35 ± 0.02	1.48 ± 0.05
MrUGT78R2	Myricetin	66.78 ± 3.56	2.31 ± 0.08	3.47 ± 0.11
	Quercetin	61.81 ± 8.74	2.76 ± 0.09	4.59 ± 0.45
	Kaempferol	58.11 ± 5.52	2.17 ± 0.05	3.78 ± 0.25
MrUGT78W1	Myricetin	13.67 ± 0.92	0.96 ± 0.05	7.02 ± 0.15
	Quercetin	93.53 ± 2.69	59.81 ± 3.20	63.90 ± 2.36
	Kaempferol	50.02 ± 4.63	34.95 ± 1.02	70.90 ± 5.71

### Transient coexpression of *MrUGTs* combined with flavonol biosynthetic genes in *Nicotiana benthamiana*

**Figure 6:**
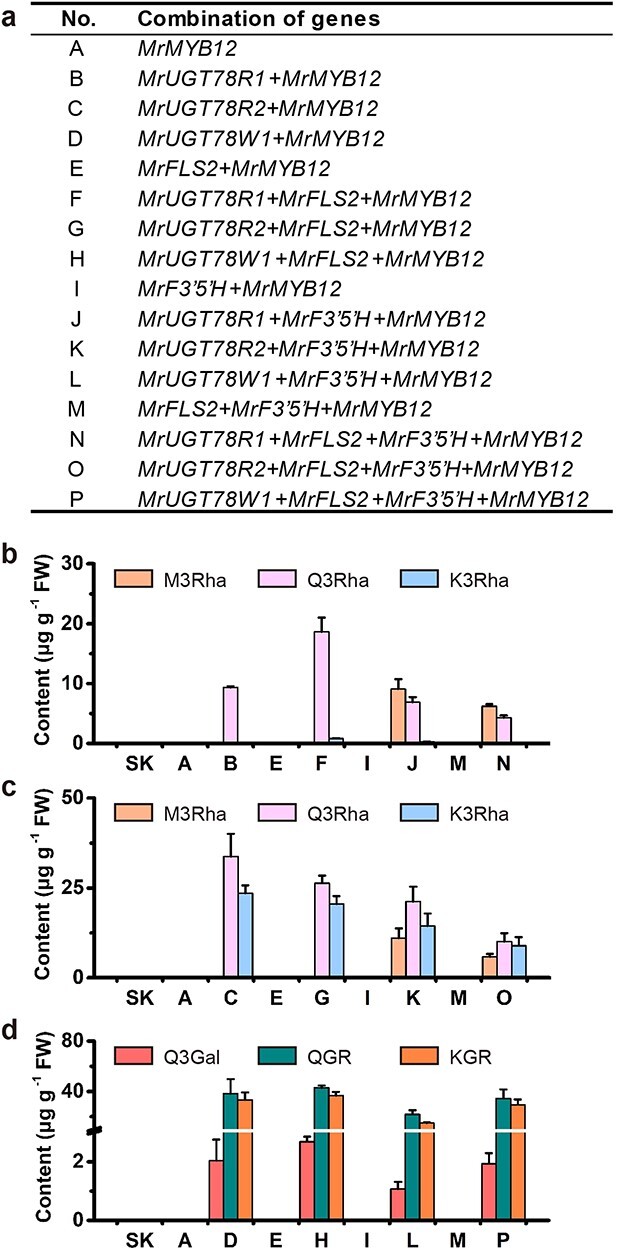
Transient expression of MrUGTs combined with flavonol biosynthesis genes in *N. benthamiana*. **a** Combinations of genes used for transient expression. **b** Flavonol rhamnoside accumulation in leaves transiently expressing combinations containing MrUGT78R1. **c** Flavonol rhamnoside accumulation in leaves transiently expressing combinations containing MrUGT78R2. **d** Flavonol galactoside accumulation in leaves transiently expressing combinations containing MrUGT78W1. Leaves injected with empty SK served as controls. Flavonol glycosides were identified by LC–MS/MS. Data are mean ± standard error (*n* = 3).

Transient expression of MrUGTs was performed in *N. benthamiana* to test the functions of MrUGTs *in vivo* according to Irmisch *et al*. [33]. *MrUGT*s in combination with flavonol biosynthetic genes *MrFLS2* and *MrF3′5′H* [24] as well as the flavonol-specific transcription factor gene *MrMYB12* [34] were tested (Fig. 6a). The results showed that up to 40 μg g^−1^ FW glycosylated flavonols could be accumulated in the leaves if the appropriate enzymes for biosynthesis of flavonols were coexpressed with any one of the three MrUGTs (Fig. 6).

*N. benthamiana* leaves accumulated abundant Q3Rha (*m*/*z* 447 with MS/MS fragment ion at *m*/*z* 300) when *MrUGT78R1* was coexpressed with *MrMYB12* (combination B) (Fig. 6b, Supplementary Data Fig. S9). Higher levels of Q3Rha and smaller amounts of K3Rha (*m*/*z* 431 with MS/MS fragment ion at *m*/*z* 285) were accumulated when *MrUGT78R1* was coexpressed with *MrFLS2* and *MrMYB12* (combination F, Fig. 6b, Supplementary Data Fig. S9). Further addition of *MrF3′5′H* (combination J, *MrUGT78R1* + *MrF3′5′H + MrMYB12*, and combination N, *MrUGT78R1* + *MrFLS2* + *MrF3′5′H + MrMYB12*) led predominantly to the formation of M3Rha (*m*/*z* 463 with MS/MS fragment ion at *m*/*z* 316) and a reduction in the formation of Q3Rha (Fig. 6b, Supplementary Data Fig. S9). As in the case of *MrUGT78R1*, *N. benthamiana* leaves accumulated high amounts of flavonol rhamnosides when infiltrated with the combinations containing *MrUGT78R2* (combinations C, G, K, and O) (Fig. 6c). No detectable level of flavonol rhamnosides accumulated in controls expressing empty vector [pGreenII0029 62_SK (SK)] or combinations without *MrUGT78R1* or *MrUGT78R2* (combinations A, E, I, M or D, H, L, and P) (Fig. 6). These results demonstrated the efficient rhamnosyl transfer activity of MrUGT78R1 and MrUGT78R2.

*N. benthamiana* leaves accumulated abundant Q3Gal (*m*/*z* 463 with MS/MS fragment ion at *m*/*z* 300) when *MrUGT78W1* was coexpressed with *MrMYB12* (combination D) (Fig. 6d, Supplementary Data Fig. S9). Two new peaks were detected in leaves that expressed combination D (Fig. 6d, Supplementary Data Fig. S9). The first peak produced by MrUGT78W1 with mother ion at *m*/*z* 609 and MS/MS fragment ion at *m*/*z* 301 was inferred to be quercetin 3-*O*-rhamnosyl galactoside (QGR) (Fig. 6d, Supplementary Data Fig. S9). Another new peak produced by MrUGT78W1 with mother ion at *m*/*z* 593 and MS/MS fragment ion at *m*/*z* 284 was inferred to be kaempferol 3-*O*-rhamnosyl galactoside (KGR) (Fig. 6d, Supplementary Data Fig. S9).

*N. benthamiana* leaves significantly accumulated Q3Gal, QGR, and KGR when infiltrated with gene combination H (*MrUGT78W1* + *MrFLS2 + MrMYB12*), combination L (*MrUGT78W1* + *MrF3′5′H + MrMYB12*), and combination P (*MrUGT78W1* + *MrFLS2 + MrF3′5′H + MrMYB12*) (Fig. 6d). No detectable level of flavonol galactosides accumulated in controls expressing SK or combinations without *MrUGT78W1* (combination A, E, I, M or B, F, J, N or C, G, K, O) (Fig. 6). These results demonstrated the efficient galactosyl transfer activity of MrUGT78W1.

## Discussion

### UGTs in the 3GT cluster contribute to the diversity of 3-OH glycosylation of flavonols

Among the ubiquitous flavonoids, there are thousands of flavonols, resulting mainly from the different types and degrees of glycosylations [8, 11, 35]. Therefore, mechanisms underlying glycosylation are of interest scientifically due to their important roles in plants physiologically and for human health. Here we identified three UGTs from *M. rubra* with different catalytic characteristics.

To date, only two 3-*O*-rhamnosyltransferases for flavonol glycosylation have been reported in plants, i.e. AtUGT78D1 from *Arabidopsis* [36] and CcUGT77B2 from montbretia (*Crocosmia × crocosmiiflora*) [35]. Here we identified the isoforms MrUGT78R1 and MrUGT78R2 as two flavonol 3-*O*-rhamnosyltransferases in *M. rubra*.

Using the *Arabidopsis ugt78d1* mutant line, UGT78D1 was identified as a flavonol-specific rhamnosyltransferase that catalyzed the rhamnosylation of Q and K at the 3-OH position [4, 36]. In montbretia, CcUGT77B2 catalyzed the transfer of rhamnose to the 3-OH position of M. The resulting M3Rha is the biosynthetic precursor of an important antidiabetic plant metabolite, montbretin A [35]. In the present study, MrUGT78R1 and MrUGT78R2 were identified as flavonol-specific 3-*O*-rhamnosyltransferases. Interestingly, *MrUGT78R1* and *MrUGT78R2* are located in proximity to one another on the same chromosome (Fig. 3b), indicating that *MrUGT78R1* and *MrUGT78R2* arose by gene duplication. Such proximal duplication of genes encoding functional rhamnosyltransferases deserves further investigation.

Several 3-*O*-galactosyltransferases with flavonol glycosylation activity have been reported in plants, i.e. F3GalTase [15], CsUGT78A15 [14], and VvGT6 [37]. F3GalTase from *Petunia hybrida* was the first identified 3-*O*-galactosyltransferase that uses only UDP-Gal and flavonol aglycones for the biosynthesis of flavonol galactosides, which are important for pollen germination [15]. In *C. sinensis*, CsUGT78A15 is a flavonol galactosyltransferase that catalyzes the biosynthesis of astringent flavonol derivatives in *C. sinensis* [14]. In grapevines, VvGT6 was identified as a bifunctional flavonol glucosyltransferase/galactosyltransferase [37]. Notably, a UDP-galactosyltransferase for flavonol glycosylation appears to be absent in *Arabidopsis*. In the present study, MrUGT78W1 was identified as a flavonol 3-*O*-galactosyltransferase and was consistently expressed in flowers and fruits of the ‘Biqi’ cultivar, where Q3Gal accumulated.

Both the abundance of precursors in plant and the enzymatic characteristics of UGT determine the type of flavonol glycosides. Enzyme kinetic analysis showed that there were small differences between the three recombinant MrUGTs tested. The much higher content of M3Rha compared with Q3Rha and Q3Gal may be due mainly to the substrate availability of MrUGTs in *M. rubra*. Recently, we reported the molecular and biochemical mechanism underlying M biosynthesis in *M. rubra*, where expression and enzyme specificity of both MrFLSs and MrF3′5′H directed the metabolic flux towards M, rather than Q [24]. In addition, the availability of sugar donors may also affect the glycosylation of flavonol, which deserves further investigation.

### Important amino acids in rhamnosyltransferase and galactosyltransferase

To date, there has been only one mutagenesis analysis carried out for rhamnosyltransferases, involving a flavonol 7-*O*-rhamnosyltransferase, AtUGT89C1, from *Arabidopsis* [38]. The last two amino acids of the PSPG box, i.e. Asp356 and His357, of AtUGT89C1 were reported as key residues for 7-*O*-rhamnosyltransferase activity since mutant D356A lost UDP-Rha recognition while mutant H357Q showed activity with both UDP-Rha and UDP-Glc [38]. Whether 3-*O*-rhamnosyltransferase activity has similar requirements for these key amino acid residues deserves further investigation. Here we investigated the effects of three alterations to the last amino acid (Asn407) of the PSPG box of MrUGT78R1, i.e. N407A, N407H, and N407Q, which all resulted in the loss of activity with UDP-Rha (Fig. 5b), indicating the importance of this residue for 3-*O*-rhamnosyltransferases. Interestingly, the flavonol rhamnosyltransferase CcUGT77B2 from montbretia has Gln (Q) as the last amino acid of the PSPG box [35] (Fig. 5a), which is similar to the mutant result of 7-*O*-rhamnosyltransferase AtUGT89C1 [38]. Our result confirmed the importance of the last amino acid residue of the PSPG box for the UDP-Rha specificity of UGT; however, rhamnosyltransferases from different plant resources have different amino acids as the last residues of the PSPG box. We provided experimental evidence that Asn (N) is important for the flavonol 3-*O*-rhamnosyltransferase of MrUGT78R1.

The penultimate amino acid of the PSPG box in known 3-*O*-rhamnosyltransferases and 7-*O*-rhamnosyltransferases is a conserved Asp (D) [35, 36, 38] (Fig. 5a). We investigated the effects of three types of mutation of this penultimate amino acid (Asp406) in the PSPG box of MrUGT78R1 (i.e. D406A, D406E, D406H) and they all resulted in the loss of rhamnosyltransfer activity (Fig. 5b), indicating the importance of this residue for catalytic activity of the 3-*O*-rhamnosyltransferases.

In addition, based on docking analysis, Pro143 and Asn386, important for rhamnosyl transfer activity, were newly identified in MrUGT78R1. Pro143 formed a hydrophobic force with the methyl group of the rhamnose moiety, which stabilized the enzyme–substrate interaction (Supplementary Data Fig. S6c), and mutation to the hydrophilic amino acid (Ser) abolished the activity of MrUGT78R1. Therefore, Pro143 is important for the recognition of UDP-Rha in MrUGT78R1. Direct interaction of Asn386 with the diphosphate group through a hydrogen bond and the loss of activity of two mutations of Asn386 demonstrated its important role in MrUGT78R1 enzyme activity (Fig. 5b, Supplementary Data Fig. S6c). In addition, mutations of Ile403, Leu404, and Gly405 resulted in the loss of enzyme activity, although these amino acids did not interact with UDP-Rha directly. They may serve to stabilize the interactions between enzyme and donor.

For UDP-galactosyltransferases, both His (H) and Gln (Q) were reported as the last amino acid residue of the PSPG box [14, 15, 39]. For example, F3GalTase [15], ACGaT [39], and CsUGT78A15 [14] contain His while MdUGT75B1 [11] and VvGT6 [37] contain Gln as the last amino acid in the PSPG box (Fig. 5a). Since UDP-glucosyltransferases usually contain Gln as the last amino acid residue of the PSPG box, UDP-galactosyltransferases, such as VvGT6, containing Gln at this position may have bifunctional activity, i.e. VvGT6 can use both UDP-Gal and UDP-Glc as sugar donor for flavonol aglycones [37]. Replacement of His374 with a Gln residue (H374Q) in ACGaT from *Aralia cordata* conferred the ability to function as a glucosyltransferase in addition to its inherent galactosyltransferase activity [39]. Similar observations were reported for another flavonol 3-*O*-galactosyltransferase, CsUGT78A15 from *C. sinensis*, where replacement of His374 with a Gln residue (H374Q) slightly reduced the activity with UDP-Gal but increased the activity with UDP-Glc [14]. Replacement of Gln373 with a His residue (Q373H) in VvGT6, however, caused a loss of ability to use UDP-Glc, without substantially affecting the activity with UDP-Gal, giving rise to a monofunctional galactosyltransferase [37]. No glucosyl transfer activity was observed for either MrUGT78W1 (Fig. 4c) or MdUGT75B1 [11]. Two types of substitution of the last amino acid residue (Gln) in the PSPG box of MrUGT78W1, i.e. Q391H and Q391N, were tested. The galactosyltransferase activity was retained when Gln was replaced with His (H) (Fig. 5b). However, replacing Gln with Asn abolished its galactosyl transfer activity (Fig. 5b), indicating the importance of His or Gln as the last amino acid in the PSPG box for the 3-*O*-galactosyltransferase.

The penultimate amino acid of the PSPG box in known 3-*O*-galactosyltransferases is conserved as Asp (D) (Fig. 5a). Interestingly, this is the same amino acid conserved as the penultimate amino acid of the PSPG box in known 3-*O*-rhamnosyltransferases [35, 36, 38]. Docking results revealed that Asp390 formed a 2.7-Å hydrogen bond with the galactose group in MrUGT78W1 while Ala390 formed a 3.5-Å hydrogen bond with the galactose group in mutant D390A of MrUGT78W1 (Supplementary Data Fig. S6d–f), which may explain the loss of galactosyl transfer activity of mutated MrUGT78W1. This indicates the importance of this residue for galactosyl transfer activity. Taken together with the mutagenesis analysis of Asp in rhamnosyltransferase (Fig. 5b), we conclude that the important role of the penultimate amino acid of the PSPG box may be conserved in both MrUGT78R1 and MrUGT78W1.

In addition, two residues (Ser147 and Asn370) important for galactosyl transfer activity were newly identified in MrUGT78W1, based on docking analysis. Asn370 in MrUGT78W1 showed a direct interaction with the diphosphate group of UDP-Gal through a hydrogen bond (Supplementary Data Fig. S6d), and results of two types of mutation for Asn370 demonstrated its important role in MrUGT78W1 enzyme activity. Unlike the presence of the hydrophobic amino acid Pro143 in MrUGT78R1, the corresponding residue in MrUGT78W1 was the hydrophilic amino acid Ser147. Ser147 formed a carbon–hydrogen bond with the galactose group (Supplementary Data Fig. S6d), and mutation to Ala generated glucosyl transfer activity in addition to the inherent galactosyl transfer activity (Fig. 5b, Supplementary Data Fig. S6). Therefore, Ser147 is important in the recognition of UDP-Gal in MrUGT78W1. Interestingly, Ser147 and Asn370 in MrUGT78W1 were the same amino acid positions as those of Pro143 and Asn386 in MrUGT78R1, respectively. Therefore, the present study identifies two amino acid positions important for both rhamnosyltransferase and galactosyltransferase.

### Exogenous expression of MrUGTs in *N. benthamiana* leaves

**Figure 7:**
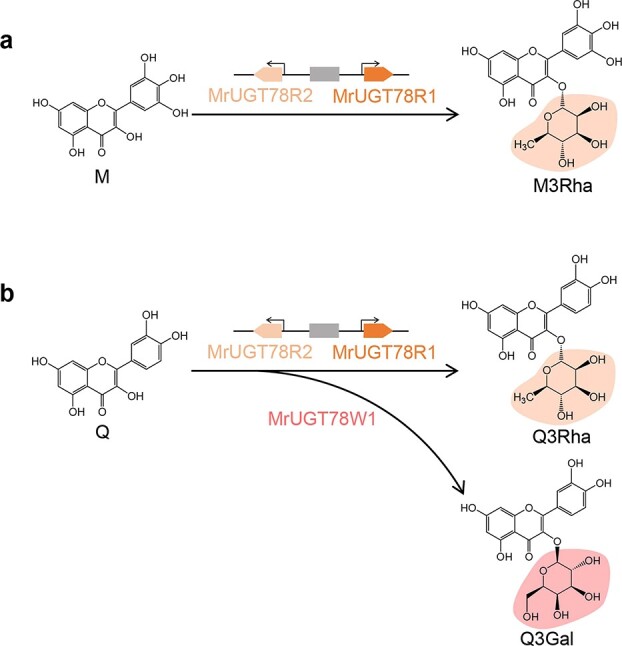
Glycosylation of flavonols in *M. rubra*. **a** MrUGT78R1 and MrUGT78R2 are involved in M3Rha biosynthesis. **b** MrUGT78R1 and MrUGT78R2 are involved in Q3Rha biosynthesis, while MrUGT78W1 is involved in Q3Gal biosynthesis.

Due to the difficulty of establishing a stable transgenic system for *M. rubra*, the function of flavonol glycosyltransferases MrUGT78R1, MrUGT78R2, and MrUGT78W1 was tested by transient expression in *N. benthamiana*. By using the flavonol-specific transcription factor gene *MrMYB12*, exogenous expression combinations with either MrUGT78R1 or MrUGT78R2 in *N. benthamiana* both accumulated high amounts of flavonol rhamnosides (M3Rha, Q3Rha, or K3Rha), while expression of combinations with MrUGT78W1 accumulated high amounts of flavonol galactosides (Q3Gal, QGR, or KGR) (Fig. 6). These transient expression data demonstrated that MrUGT78R1 and MrUGT78R2 function as UDP-rhamnosyltransferases involved in flavonol rhamnosylation (Fig. 7a) and that MrUGT78W1 functions as a UDP-galactosyltransferase involved in flavonol galactosylation (Fig. 7b), although their *in vivo* function in flavonol glycosylation should be further tested in its natural host, *M. rubra*, when its transgenic system is available.

*N. benthamiana* could serve as a plant host for metabolic engineering to produce plant metabolites with complex structure. However, heterologous plant systems for metabolic engineering may have a problem, which is the potential production of non-target compounds, while overexpressing transcription factors that control specific metabolite pathways could direct the flux to the target pathways and enhance production levels [40, 41]. For example, in the present study, by coexpressing with the flavonol-specific transcription factor MrMYB12, large amounts of target flavonol rhamnosides and galactosides were produced in *N. benthamiana*. In addition, by transiently expressing five CcUGTs combined with CcMYB4 and other flavonol biosynthesis genes from montbretia in *N. benthamiana*, Irmisch *et al*. [42] achieved production of the antidiabetic plant metabolite montbretin A. These results together with the present study demonstrated the potential for metabolic engineering of flavonols with specific glycosylation by ectopic expression of UGT genes in plants.

In summary, by both *in vitro* and *in vivo* studies, MrUGT78R1 and MrUGT78R2 were identified as UDP-rhamnosyltransferases, while MrUGT78W1 was identified as a UDP-galactosyltransferase. Pro143 and Asn386 were identified as important residues for rhamnosyl transfer activity in MrUGT78R1, while these two corresponding positions in MrUGT78W1 (i.e. Ser147 and Asn370) also play important roles in galactosyl transfer activity. This work provides better understanding of the involvement of UDP-rhamnosyltransferase and UDP-galactosyltransferase in flavonol glycosylation, which may assist in glycosylation modification of bioactive compounds with diverse health-promoting benefits.

## Materials and methods

### Chemicals and reagents

M, Q, K, M3Rha, Q3Rha, Q3Gal, K3Gal, K3Rha, flavanols (catechin and epicatechin), flavanones (naringenin and hesperetin), isoflavones (genistein and daidzein), and flavones (apigenin and luteolin) were all HPLC standard and obtained from Aladdin (Shanghai, China). UDP-Rha was bought from Yuanye (Shanghai, China). HPLC-grade acetonitrile and methanol, UDP-Glc, and UDP-Gal were bought from Sigma–Aldrich (St Louis, MO, USA).

### Plant materials

Flowers, leaves, and fruits of two *M. rubra* cultivars, ‘Biqi’ and ‘Dongkui’, were harvested from Lanxi, Zhejiang province, China. S1, S2, S3 and S4 represent four fruit development stages: 45, 75, 80, and 85 days after flowering. Samples were selected for uniformity and absence of mechanical damage. Samples were cut and frozen in liquid nitrogen before being stored at −80°C. All experiments were carried out with at least three biological replicates.

### Flavonol glycoside analysis by HPLC

Analysis of flavonol glycosides was performed as previously reported [43]. Sample powder was extracted with 50% aqueous methanol (material:solvent ratio of 1:10) by sonification for 30 minutes, twice, and supernatants were combined after centrifugation at 12 000 rpm for 15 minutes. Supernatants were analyzed by HPLC (2998 PDA detector, e2695 pump, Waters, USA). Flavonol glycosides were identified at 350 nm and quantified by comparison with authentic standards.

### *MrUGT* gene identification and phylogenetic analysis

A UGT hidden Markov model (HMM) profile (PF00201) obtained from the online protein database (http://pfam.xfam.org/) was used to identify MrUGT by the Simple HMM Search program in TBtools [31, 44]. Conserved Domain Search in NCBI and Multiple EM for Motif Elicitation (MEME, suite 5.0.3) were used to confirm the completeness of the conserved PSPG box with the help of TBtools [44]. MEME analysis was performed with default parameters. Incomplete full-length coding sequences were manually corrected using data from the RNA-Seq database (PRJNA714192). Names of MrUGTs were suggested by the UGT Nomenclature Committee (https://prime.vetmed.wsu.edu/resources). The accession number of MrUGT78W1 was updated to MZ727195. Protein sequences of *M. rubra* and other known UGTs were aligned using the MUSCLE program followed by constructing a maximum likelihood tree with 1000 bootstrap replicates in MEGA-X software. Protein sequences of MrUGTs were aligned with other known UGTs by the MUSCLE program in MEGA-X software using default parameters, and then the alignment was visualized by GeneDoc software. GenBank numbers of identified UGTs are presented in Supplementary Data Table S1.

### Gene expression analysis

RNA was isolated using the CTAB method from ‘Biqi’ and ‘Dongkui’ cultivars as reported [30]. HiScript II 1st Strand cDNA Synthesis Kit (+gDNA wiper) was used for cDNA synthesis according to the manual (Vazyme Biotech Co. Ltd, Nanjing, China). qRT–PCR was carried out with specific primers (Supplementary Data Table S2) according to a previous report [43]. Gene expression was calculated by the 2^-∆t^ method with the *actin* gene (*MrACT*, GQ340770) as internal reference gene.

### Expression and purification of recombinant MrUGT proteins

Recombinant protein was expressed according to our previous report with modifications [11]. The coding sequence of each *MrUGT* was subcloned into pET-32a(+) vector using the primers shown in Supplementary Data Table S3. After confirmation by sequencing, positive plasmids were transformed into BL21(DE3)pLysS-competent cells (TransGen Biotech, Beijing, China). Cultures were incubated at 37°C in Luria–Bertani broth medium (ampicillin, 100 mg L^−1^) to OD_600_ of 0.6–0.8. Recombinant protein was induced by 0.5 mM isopropyl β-d-thiogalactoside (IPTG) and cultured for 20 hours at 16°C. Cells were collected by centrifugation and resuspended with extraction buffer. After freezing overnight at −80°C, cells were disrupted by sonification. Protein was obtained by centrifugation (10 000 rpm, 30 minutes, at 4°C) and purified by HisTALON Gravity Columns (Takara Bio Inc., Beijing, China). PD-10 columns (GE Healthcare, UK) were used for protein desalting according to the manual before SDS–PAGE analysis (Supplementary Data Fig. S2). Protein was quantified by using a BCA assay kit (FUDE Biotech, Hangzhou, China).

### Enzymatic activity assay

Initial enzymatic assay was performed as previously reported [11]. Enzyme activities were carried out in reaction mixture (100 μL) containing Tris–HCl buffer (100 mM, pH 7.5, 2.0 mM dithiothreitol), recombinant MrUGT protein (2.0–2.5 μg), 1 mM sugar donors (UDP-Rha/UDP-Gal/UDP-Glc), and 500 μM acceptors at 30°C for 20 minutes. An equal volume of methanol was added to the mixture to stop enzyme assays. Enzyme products were analyzed by HPLC at 350 nm. Mobile phases consisted of aqueous formic acid (0.1%, v/v, eluent A) and acetonitrile with formic acid (0.1%, v/v, eluent B). The elution program was 10–50% B for 0–7minutes; 50% B for 7–10 minutes; 50–100% B for 10–15 minutes; 100–10% B for 15–16 minutes; 10% B for 16–20 minutes. Products were confirmed by LC–MS/MS using a triple quadrupole mass spectrometer (Agilent 6460) equipped with an ESI source and an Agilent ZORBAX SB-C18 column (250 × 4.6 mm, 5 μm) (Agilent Technologies, Santa Clara, CA, USA). Mass spectrometry data were analyzed in negative ionization mode.

The optimum reaction pH for recombinant MrUGT proteins was investigated using different buffer solutions over the range of pH 4.0–11.0 at 30°C for 20 minutes, with further analysis by HPLC. Incubation buffers included sodium citrate buffer (100 mM, pH 4.0–6.0, buffer A), phosphate buffer (100 mM, pH 5.5–8.0, buffer B), Tris–HCl buffer (100 mM, pH 7.5–9.5, buffer C), and Na_2_CO_3_/NaHCO_3_ buffer (100 mM, pH 8.5–11.0, buffer D). The optimum temperature was investigated over the range of 10 to 60°C at the optimum pH investigated above for 20 minutes. Different flavonoids, including flavones, flavanols, flavanones, and isoflavones, were used to analyze the acceptor specificity of MrUGTs. Products of flavanols, flavanones, and isoflavones were detected at 280 nm, while products of flavones were detected at 350 nm. When investigating optimum conditions of enzyme activity and acceptor specificity, proteins were expressed in at least three different batches and then purified.

The UDP-Glo™ Glycosyltransferase Assay Kit [45] was used for kinetic analysis. Production of UDP in the glycosylation reaction was converted to ATP by the kit, and was detected with a microplate reader (Synergy H1, BioTek); luminescence was correlated to UDP concentration by using an UDP standard curve. For MrUGT78R1, recombinant proteins (1.0 μg) were analyzed in reaction mixtures (50 μL) containing 650 μM UDP-Rha in phosphate buffer (100 mM, pH 6.0) at 50°C for 20 minutes. For MrUGT78R2, recombinant proteins (1.0 μg) were analyzed in reaction mixtures (50 μL) containing 650 μM UDP-Rha in Tris–HCl buffer (100 mM, pH 7.5) at 35°C for 20 minutes. For MrUGT78W1, recombinant proteins (1.0 μg) were analyzed in reaction mixtures (50 μL) containing 650 μM UDP-Gal in Tris–HCl buffer (100 mM, pH 8.5) at 30°C for 20 minutes. Enzymatic reaction mixtures (25 μL) were transferred to a 96-well assay plate and the reaction was stopped by adding 25 μL of UDP Detection Reagent to each well. The concentration range of flavonol aglycones was 0–300 μM. *K*_m_ and *V*_max_ were calculated by non-linear curve fitting of the Michaelis–Menten function in Origin (version 9.0) from at least three different batches of proteins.

### Molecular docking analysis

Homology 3D models of MrUGT78R1 and MrUGT78W1 were established using SWISS-MODEL based on the template of VvGT1 (PDB ID: 2C1X). UDP-Rha and UDP-Gal were docked into the active site of MrUGT78R1 and MrUGT78W1, respectively, using Autodock Vina [46]. Models were viewed and rendered using PyMOL (version 2.3.4).

### Site-directed mutagenesis analysis

Multiple protein sequence alignment was performed between MrUGTs and other known UGTs as mentioned above. Site-directed mutagenesis was performed by overlapping PCR. Mutated sequences were cloned into pET expression vector and verified by sequencing. Primers for mutagenesis are shown in Supplementary Data Table S3. The reaction mixture (100 μL) for mutant enzyme assay contained Tris–HCl buffer (100 mM, pH 7.5), 500 μM Q, 1 mM sugar donors (UDP-Rha/UDP-Gal/UDP-Glc), and 30–40 μg crude mutant protein. Products were analyzed by HPLC.

### Transient coexpression of *MrUGT*s combined with flavonol biosynthetic genes in *N. benthamiana*

Transient expression was performed in *N. benthamiana* to test the functions of *MrUGT*s *in vivo* according to a previous report [43]. Coding sequences of *MrUGT78R1*, *MrUGT78R2*, or *MrUGT78W1* were cloned into pGreenII0029 62_SK (SK) vector using primers shown in Supplementary Data Table S4. Positive plasmids were transformed into *Agrobacterium tumefaciens* GV3101 by electroporation. After positive strain examination, *A. tumefaciens* cells were resuspended in infiltration buffer (pH 5.6, 10 mM MES, 10 mM MgCl_2_, 150 μM acetosyringone) to OD_600_ = 0.75. Combination information is shown in Fig. 6a. Each combination contained equal proportions of transformed *A. tumefaciens* and p19 strain. Four-week-old *N. benthamiana* leaves were injected with different gene combinations. Injected leaves were harvested and analyzed 5 days after infiltration. Leaves injected with empty SK served as control. Flavonol glycosides were analyzed by LC–MS/MS (Supplementary Data Fig. S9). Data for each combination were obtained from at least three biological replicates.

### Statistical analysis

Chemical structures were drawn by ChemDraw (version 20.0, PerkinElmer, Waltham, MA, USA). Raw data were analyzed and presented by Origin software (version 9.0, OriginLab, Northampton, MA, USA). Data for all experiments were presented with mean ± standard error from at least three biological replicates.

## Acknowledgements

This work was financially supported by the National Natural Science Foundation of China (31872067), the Key Research & Development Program of Zhejiang Province (2021C02001), and the 111 project (B17039). We thank Prof. Liang Yan for providing *A. tumefaciens* p19 strain.

## Author contributions

X.L. designed the research plan. C.H.R. performed the experiments with the help of Y.G., L.F.X., Z.K.Z., M.Y.X., Y.L.C., Y.L.L., and J.L. B.Z., C.J.X., and K.S.C. supported the *M. rubra* project. X.L. and C.H.R. drafted this manuscript. D.G. contributed to discussions, revisions, and language enhancement. The article was approved by all authors.

## Data availability

GenBank accession numbers for MrUGT78R1, MrUGT78R2, and MrUGT78W1 were KAB1224450, KAB1224448, and MZ727195, respectively. The BioProject accession for RNA-Seq data of ‘Biqi’ was PRJNA714192.

## Conflict of interest

There is no conflict of interest to declare.

## Supplementary data

Supplementary data is available at *Horticulture Research* online.

## Supplementary Material

supp_data_uhac138
