# Supplementary material for: Identification of UDP-rhamnosyltransferases and UDP-galactosyltransferase involved in flavonol glycosylation in Morella rubra
Source: Hortic Res. 2022 Jun 20;9:uhac138. doi: 10.1093/hr/uhac138 (PMC9437722; doi:10.1093/hr/uhac138)
Supplement: supp_data_uhac138 [file supp_data_uhac138.zip › 04 Revised Supporting Information HR-2022-192 marked-up.docx]

**
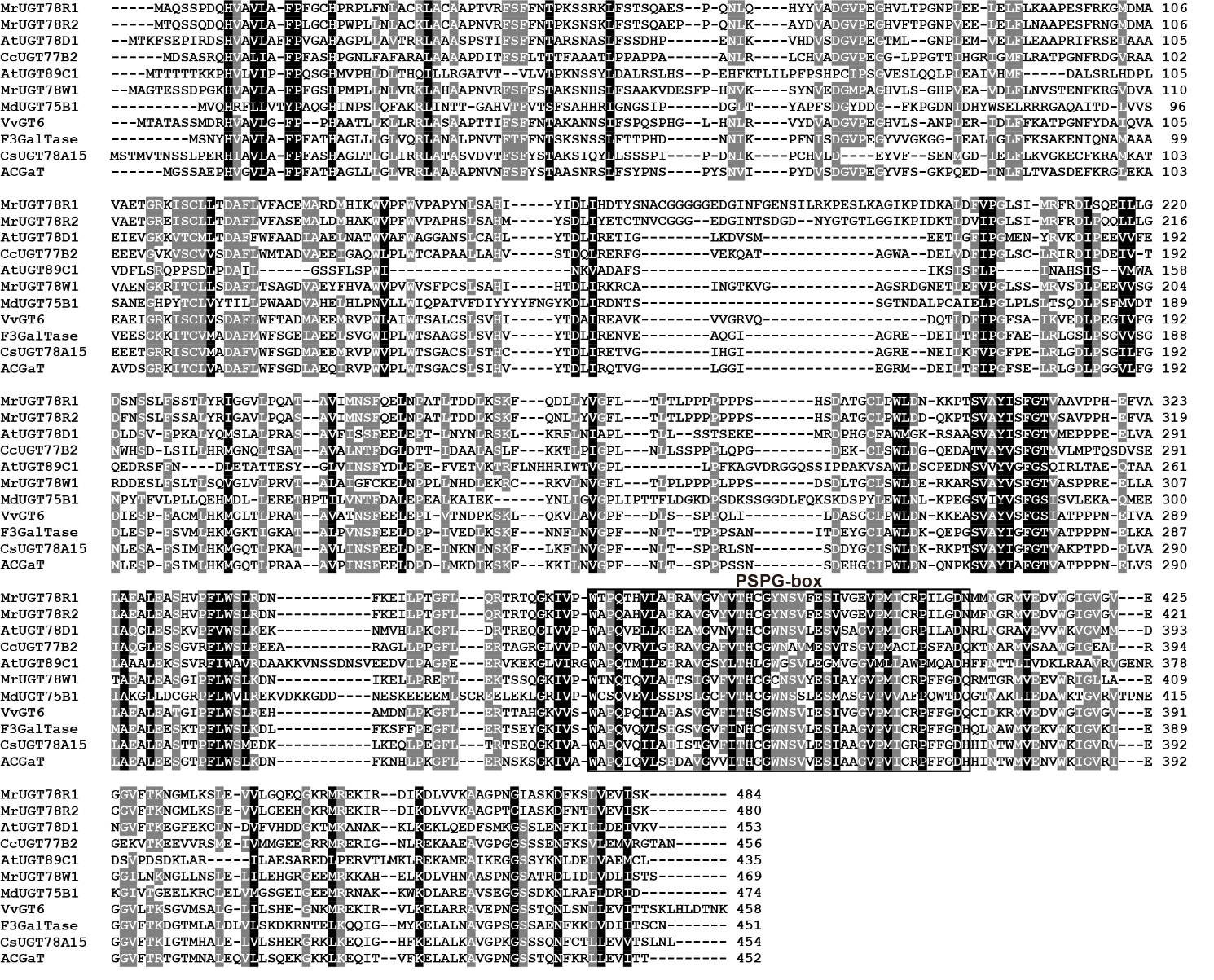
**

**Figure S1.** Multiple sequence alignment of MrUGTs with other known UGTs. Fully conserved residues are shown in white characters boxed in black shading. Similar residues are shown in white bold characters and boxed with gray shading. The conserved PSPG-box are boxed in black.

**
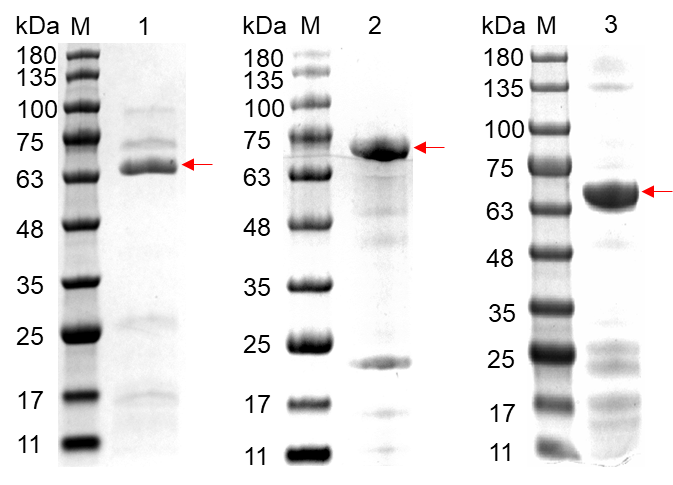
**

**Figure S2.** SDS-PAGE analysis of the His-tagged MrUGT78R1, MrUGT78R2, and MrUGT78W1 proteins. Lane 1, MrUGT78R1; Lane 2, MrUGT78R2; Lane 3, MrUGT78W1; Lane M, protein molecular weight marker. Red arrows indicate recombinant proteins.

**
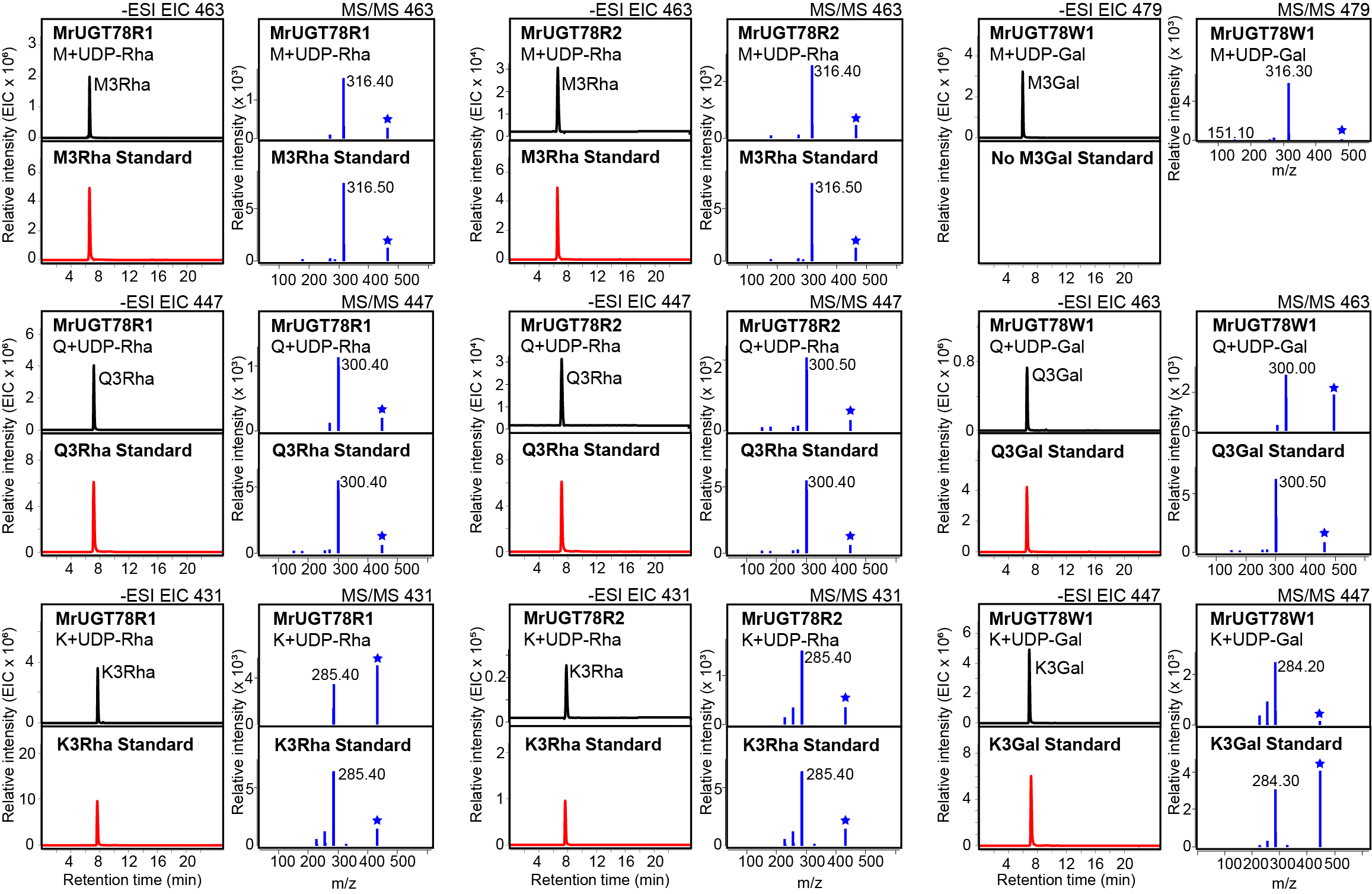
**

**Figure S3.** LC-MS/MS analysis of the products generated by MrUGT78R1, MrUGT78R2, and MrUGT78W1 when supplied with UDP-Rha or UDP-Gal and different flavonol aglycones. Extract ion chromatograms (EIC) and mass spectrum information are shown. Asterisks indicate mother ion for MS/MS fragmentation.

**
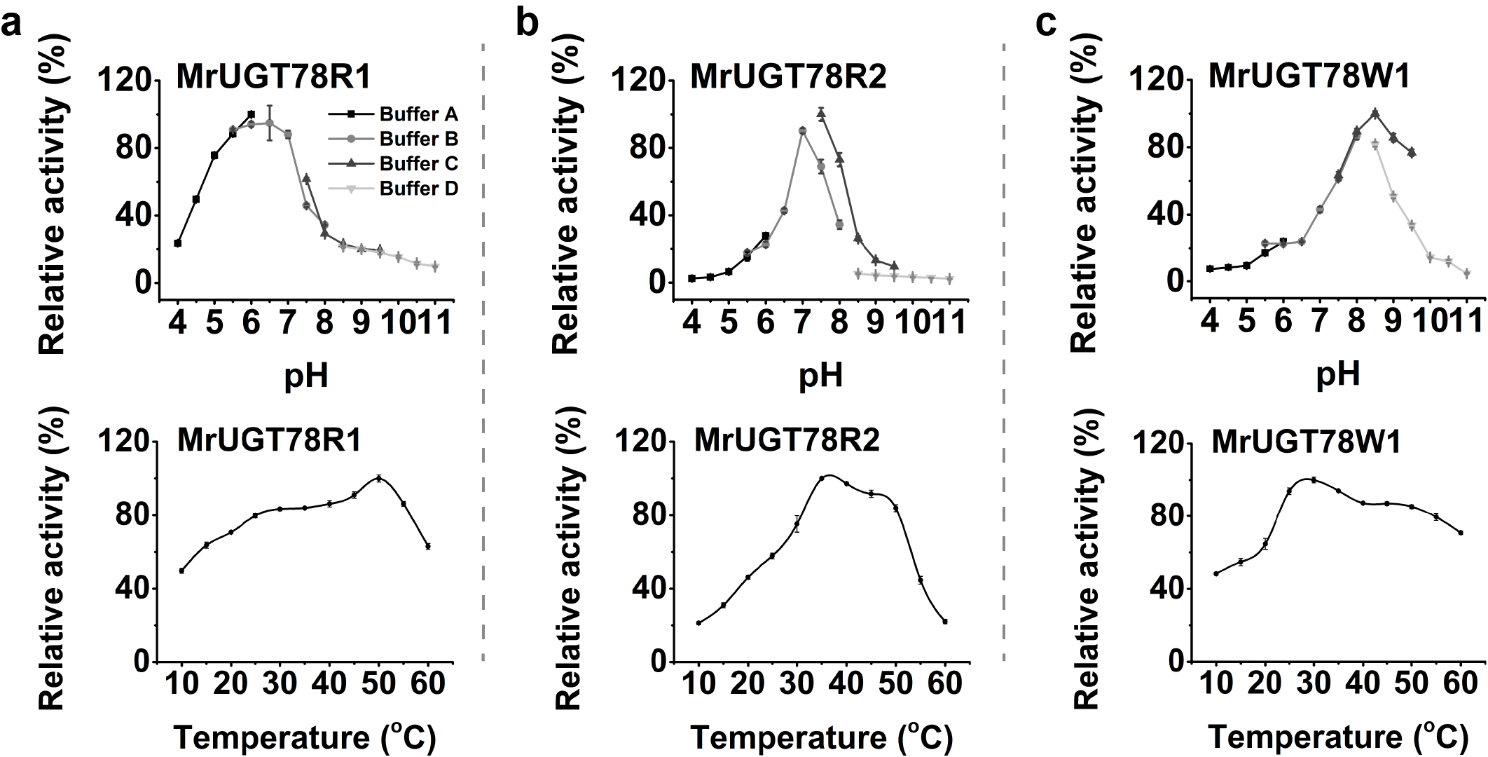
**

**Figure S4.** Optimum reaction conditions for recombinant MrUGT78R1 **a**, MrUGT78R2 **b**, and MrUGT78W1 **c**. Buffer A, sodium citrate buffer; Buffer B, phosphate buffer; Buffer C, Tris-HCl buffer; Buffer D, Na_2_CO_3_/NaHCO_3_ buffer.**
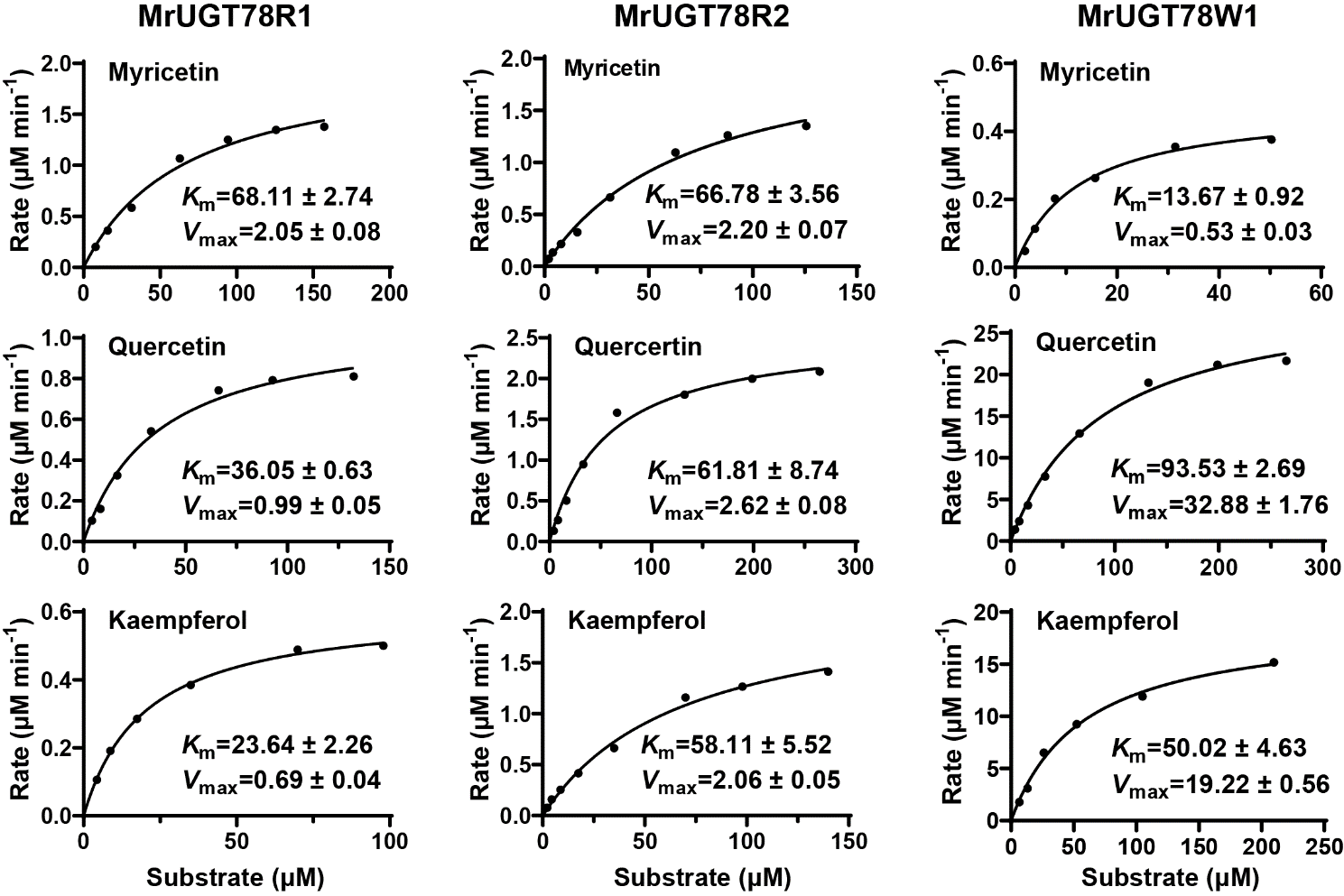
**

**Figure S5.** Michaelis-Menten plots of recombinant MrUGT78R1, MrUGT78R2, and MrUGT78W1.

**
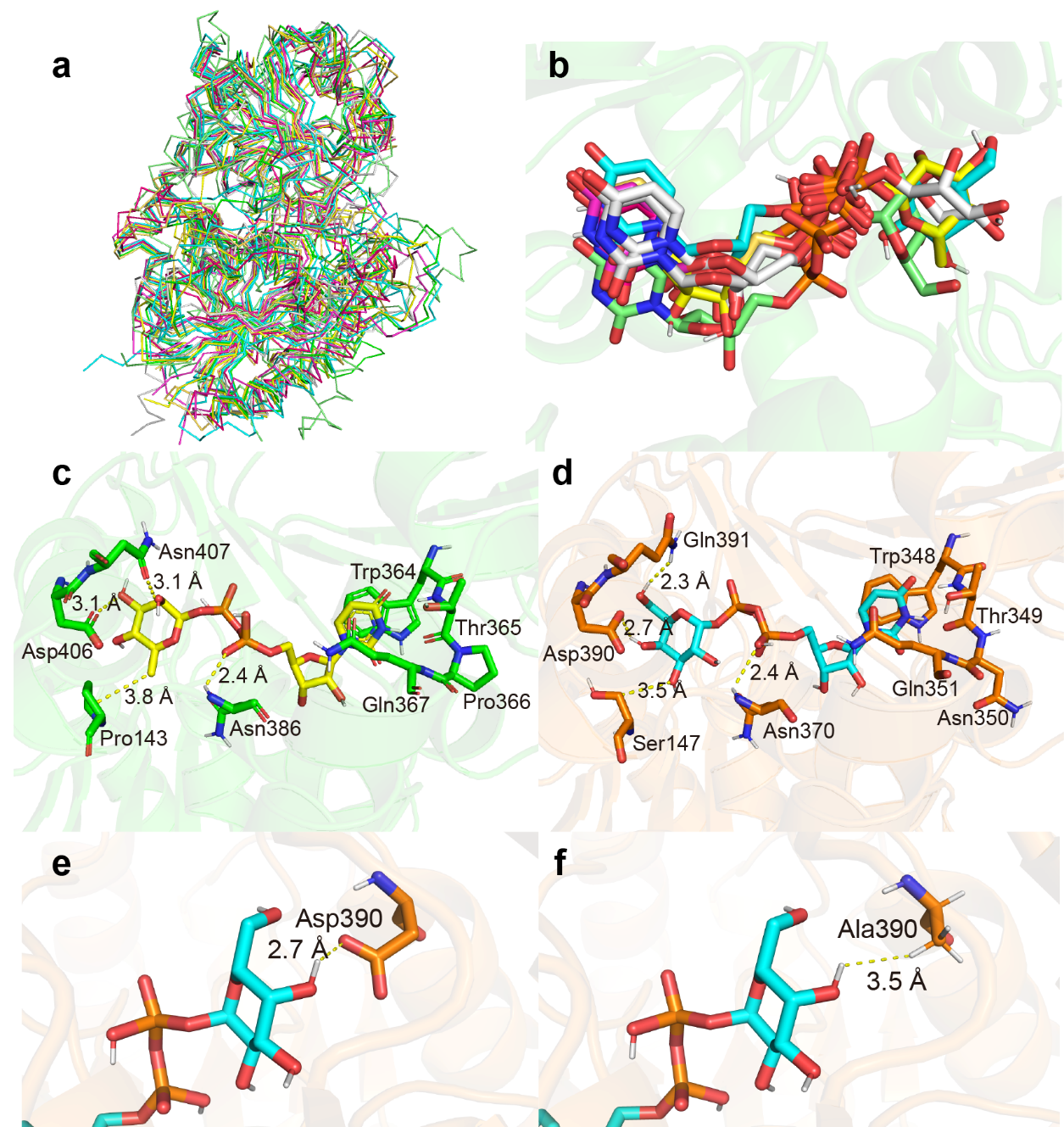
**

**Figure S6.** Molecular docking analysis of MrUGT78R1 and MrUGT78W1. **a** Structure alignment of UGTs. All structures are showed with ribbon and superposed by PyMOL. The PDB numbers of these structures are 6IJA, 2C1X, 3HBF, 2VG8, 5GL5, 5TMB, and 5U6M. **b** Ligands in superposed structures are showed with sticks. **c** Docking analysis important residues for MrUGT78R1. **d** Docking analysis important residues for MrUGT78W1. Docking analysis of Asp390 (e) and mutant D390A (f) of MrUGT78W1.

**
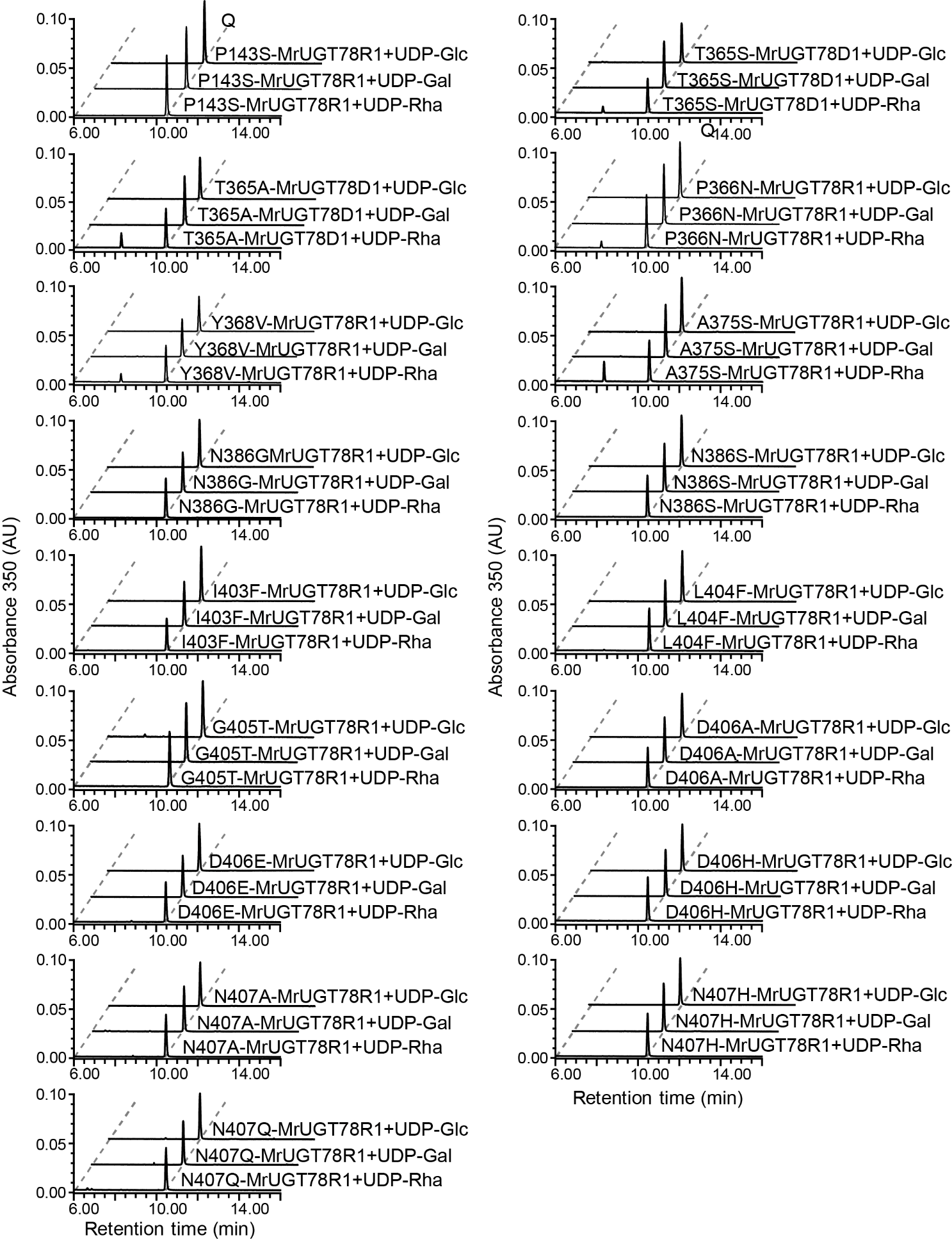
**

**Figure S7.** HPLC analysis the enzymes activity for mutation proteins of MrUGT78R1 with quercetin as sugar acceptor.

**
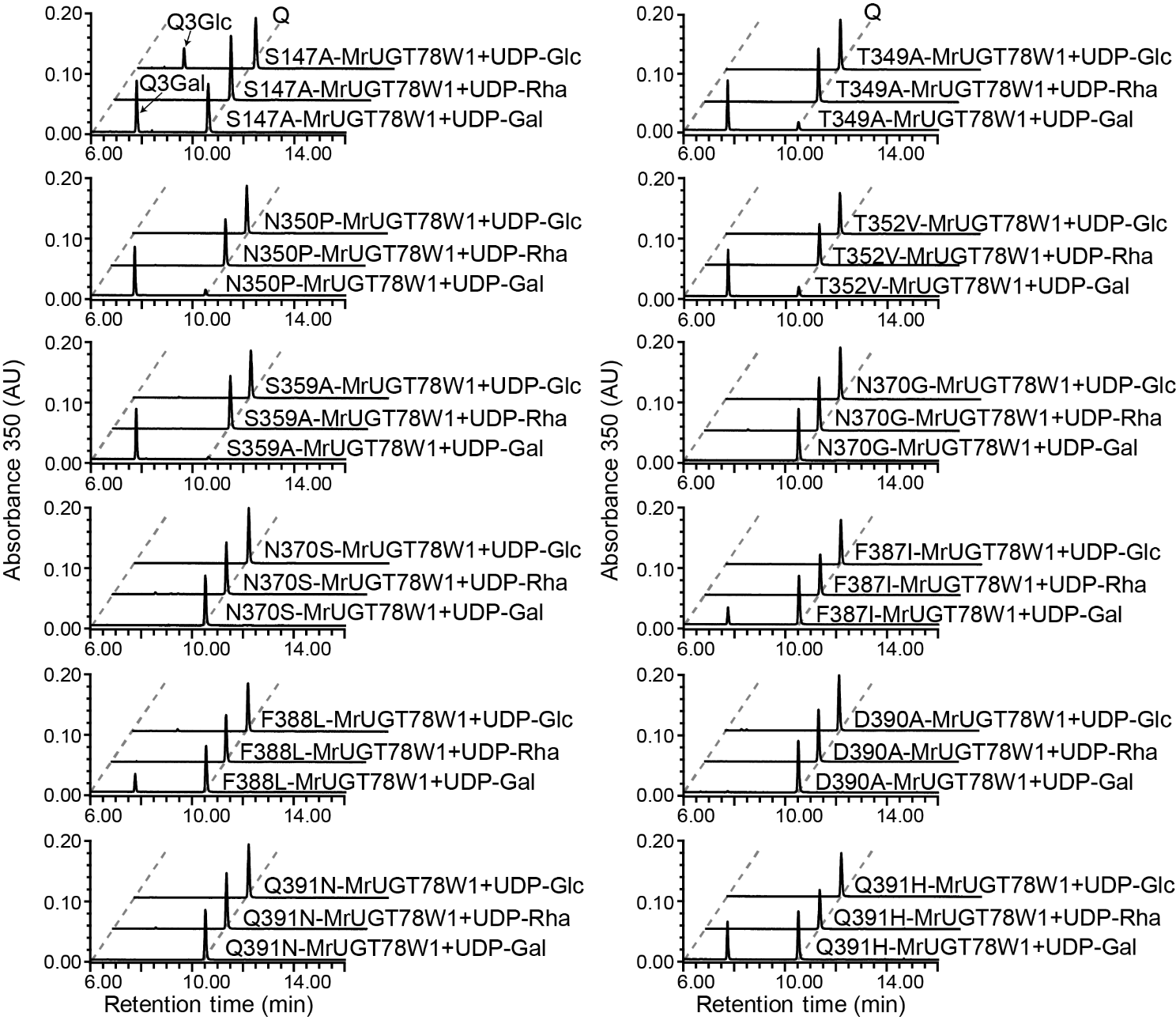
**

**Figure S8**. HPLC analysis the enzymes activity for mutation proteins of MrUGT78W1 with quercetin as sugar acceptor.

**
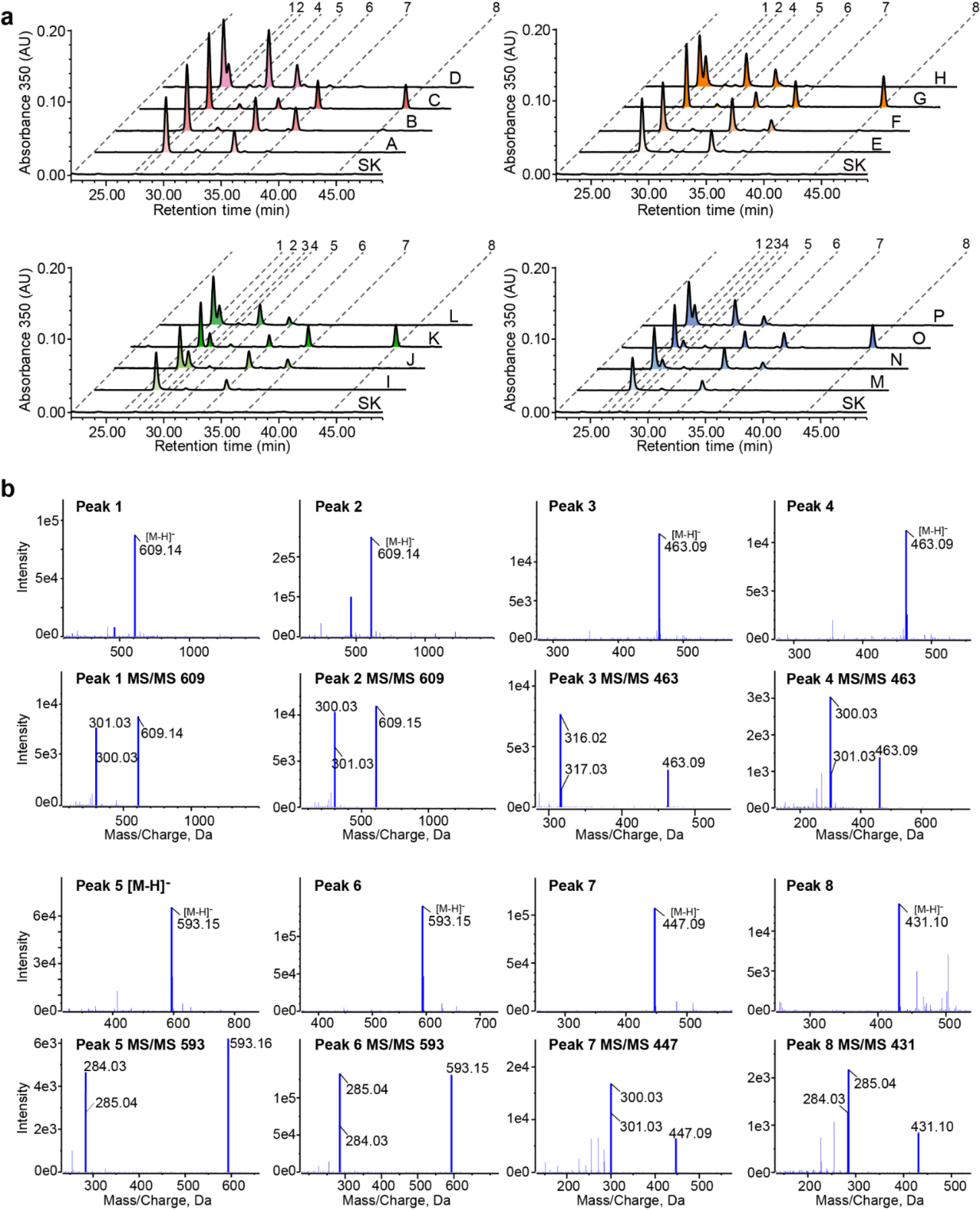
**

**Figure S9.** Identification of products produced by transient coexpression of MrUGTs with flavonol biosynthesis pathway genes in *Nicotiana benthamiana*.

**a,** HPLC chromatograms of *N. benthamiana* leaves infiltrated with gene combinations. **b,** Flavonol glycosides accumulated in *N. benthamiana* leaves were identified by LC-MS/MS. Fragmentation information were shown.

Peak 1, quercetin rhamnosyl-galactoside (QGR); Peak 2, quercetin 3-*O*-rutinoside (Q3Rut); Peak 3, myricetin 3-*O*-rhamnoside (M3Rha); Peak 4, quercetin 3-*O*-galactoside (Q3Gal); Peak 5, kaempferol rhamnosyl-galactoside (KGR); Peak 6, kaempferol 3-*O*-rutinoside (K3Rut); Peak 7, quercetin 3-*O*-rhamnoside (Q3Rha); Peak 8, kaempferol 3-*O*-rhamnoside (K3Rha).

**Table S1.** Genbank accession numbers of identified UGTs in the phylogenetic tree.

| **Gene** | **Accession number** | **Species** | |
| --- | --- | --- | --- |
| AtUGT73C6 | At2g36790 | Arabidopsis | |
| AtUGT75C1 | At4g14090 | Arabidopsis | |
| AtUGT78D1 | At1g30530 | Arabidopsis | |
| AtUGT78D2 | At5g17050 | Arabidopsis | |
| AtUGT78D3 | At5g17030 | Arabidopsis | |
| AtUGT79B1 | At5g54060 | Arabidopsis | |
| AtUGT79B2 | At4g27560 | Arabidopsis | |
| AtUGT79B3 | At4g27570 | Arabidopsis | |
| AtUGT79B6 | At5g54010 | Arabidopsis | |
| AtUGT89C1 | At1g06000 | Arabidopsis | |
| ACGT | AB103471 | *Aralia cordata* | |
| CsUGT78A14 | KP682360 | *Camellia sinensis* | |
| CsUGT78A15 | KP682361 | *Camellia sinensis* | |
| CcUGT77B2 | MG938542 | *Crocosmia × crocosmiiflora* | |
| DkFGT | AB435084 | *Diospyros kaki* | |
| FaGT1 | AAU09442 | *Fragaria × ananassa* | |
| FaGT7 | ABB92749 | *Fragaria × ananassa* | |
| Gt5GT7 | BAG32255 | *Gentiana triflora* | |
| GeUGT73F1 | AB098614 | *Glycyrrhiza echinata* | |
| Ib3GGT | EF108571 | *Ipomoea batatas* | |
| Iris5GT | BAD06874 | *Iris hollandica* | |
| LeABRT2 | LC131336 | *Lobelia erinus* | |
| LeABRT4 | LC131337 | *Lobelia erinus* | |
| MdUGT75B1 | MDP0000545122 | *Malus × domestica* | |
| F3GalTase | AAD55985 | *Petunia hybrida* | |
| Ph5GT | AB027455 | *Petunia hybrida* | |
| Ph3RT | X71059 | *Petunia hybrida* | |
| PhUGT79B31 | LC387490 | *Petunia hybrida* |  |
| PpUGT78B | ppa005162m | *Prunus persica* | |
| UBGT | AB031274 | *Scutellaria baicalensis* | |
| Va5GT | KF996717 | *Vitis amurensis* | |
| VvGT1 | AB047092 | *Vitis vinifera* | |
| VvGT5 | AB499074 | *Vitis vinifera* | |
| VvGT6 | AB499075 | *Vitis vinifera* | |

**Table S2.** Primers for cloning *MrUGT78R1*, *MrUGT78R2*, and *MrUGT78W1* and for qRT-PCR.

| **Gene** | **Primer** | **Sequence** |
| --- | --- | --- |
| MrUGT78R1 | Forward | ATGGCCCAAAGCTCGCCGGA |
|  | Reverse | TTATTTGGAGATCACCTCTACCAAAC |
| MrUGT78R2 | Forward | ATGACACAAAGCTCGCAGGA |
|  | Reverse | TTATTTGGAAATTACCTCCACCAATGTAT |
| MrUGT78W1 | Forward | ATGGCTGGGACGGAAAGTTCTGAT |
|  | Reverse | TTAAGACGTAGATATTAAGTCCACCAG |
| qPCR-MrUGT78R1 | Forward | GTAGGCCGATCTTGGGTGATAAT |
|  | Reverse | TTTGGAAGCAATCCCATTTGGTC |
| qPCR-MrUGT78R2 | Forward | TTGGAACGGTGTCAGCAGTT |
|  | Reverse | AAGACATGAGCCTGAGGTGC |
| qPCR-MrUGT78W1 | Forward | CTAGGTCCGTCGCATATGTCAGC |
|  | Reverse | TCCCTTGCGAGCTTGTCTTTTCA |

**Table S3.** Primers for recombinant proteins expression and site-directed mutagenesis. Restriction sites are underlined.

| **Gene** | **Sequence** |
| --- | --- |
| MrUGT78R1 F-pET | gccatggctgatatcGGATCCATGGCCCAAAGCTCGCCGGA |
| MrUGT78R1 R-pET | gtggtggtggtggtgCTCGAGTTTGGAGATCACCTCTACCAAAC |
| MrUGT78R2 F-pET | gccatggctgatatcGGATCCATGACACAAAGCTCGCAGGA |
| MrUGT78R2 R-pET | gtggtggtggtggtgCTCGAGTTTGGAAATTACCTCCACCAATGTAT |
| MrUGT78W1 F-pET | gccatggctgatatcGGATCCATGGCTGGGACGGAAAGTTCTGAT |
| MrUGT78W1 R-pET | gtggtggtggtggtgCTCGAGAGACGTAGATATTAAGTCCACCAG |
| MrUGT78R1 P143S R1 | GTTGTAGGGTGCAGAAACCCAAAAAGGAAC |
| MrUGT78R1 P143S F1 | GTTCCTTTTTGGGTTTCTGCACCCTACAAC |
| MrUGT78R1 T365S R1 | ATGAGTCTGGGGTGACCATGGAACTATTTTTCCCT |
| MrUGT78R1 T365S F1 | AGGGAAAAATAGTTCCATGGTCACCCCAGACTCAT |
| MrUGT78R1 T365A R1 | ATGAGTCTGGGGTGCCCATGGAACTATTTTTCCCT |
| MrUGT78R1 T365A F1 | AGGGAAAAATAGTTCCATGGGCACCCCAGACTCAT |
| MrUGT78R1 P366N R1 | GACATGAGTCTGATTTGTCCATGGAACTAT |
| MrUGT78R1 P366N F1 | ATAGTTCCATGGACAAATCAGACTCATGTC |
| MrUGT78R1 Y368V R1 | GTGTGCCAAGACTTGAGTCTGGGGTGTCCATGG |
| MrUGT78R1 Y368V F1 | CCATGGACACCCCAGACTCAAGTCTTGGCACAC |
| MrUGT78R1 A375S R1 | CACATACACGCCTACGCTCCTGTGTGCCAAGAC |
| MrUGT78R1 A375S F1 | GTCTTGGCACACAGGAGCGTAGGCGTGTATGTG |
| MrUGT78R1 N386G R1 | GCTCTCAAACACGGAGCCGTATCCACAGTGGGTCAC |
| MrUGT78R1 N386G F1 | GTGACCCACTGTGGATACGGCTCCGTGTTTGAGAGC |
| MrUGT78R1 N386S R1 | GCTCTCAAACACGGATGAGTATCCACAGTGGGTCAC |
| MrUGT78R1 N386S F1 | GTGACCCACTGTGGATACTCATCCGTGTTTGAGAGC |
| MrUGT78R1 I403F R1 | CATATTATCACCCAAAAACGGCCTACAGAT |
| MrUGT78R1 I403F F1 | ATCTGTAGGCCGTTTTTGGGTGATAATATG |
| MrUGT78R1 L404F R1 | CATATTATCACCAAAGATCGGCCTACAGAT |
| MrUGT78R1 L404F F1 | ATCTGTAGGCCGATCTTTGGTGATAATATG |
| MrUGT78R1 G405T R1 | CATATTATCAGTCAAGATCGGCCTACAGAT |
| MrUGT78R1 G405T F1 | ATCTGTAGGCCGATCTTGACTGATAATATG |
| MrUGT78R1 D406A R1 | ACCATCCGTCCGTTCATCATATTGGCACCCAAGATCGGCC |
| MrUGT78R1 D406A F1 | GGCCGATCTTGGGTGCCAATATGATGAACGGACGGATGGT |
| MrUGT78R1 D406E R1 | ACCATCCGTCCGTTCATCATATTCTCACCCAAGATCGGCC |
| MrUGT78R1 D406E F1 | GGCCGATCTTGGGTGAGAATATGATGAACGGACGGATGGT |
| MrUGT78R1 D406H R1 | ACCATCCGTCCGTTCATCATATTGTGACCCAAGATCGGCC |
| MrUGT78R1 D406H F1 | GGCCGATCTTGGGTCACAATATGATGAACGGACGGATGGT |
| MrUGT78R1 N407A R1 | ACCATCCGTCCGTTCATCATGGCATCACCCAAGATCGGCC |
| MrUGT78R1 N407A F1 | GGCCGATCTTGGGTGATGCCATGATGAACGGACGGATGGT |
| MrUGT78R1 N407H R1 | ACCATCCGTCCGTTCATCATGTGATCACCCAAGATCGGCC |
| MrUGT78R1 N407H F1 | GGCCGATCTTGGGTGATCACATGATGAACGGACGGATGGT |
| MrUGT78R1 N407Q R1 | ACCATCCGTCCGTTCATCATTTGATCACCCAAGATCGGCC |
| MrUGT78R1 N407Q F1 | GGCCGATCTTGGGTGATCAAATGATGAACGGACGGATGGT |
| **Table S3 Continued** |  |
| **Gene** | **Sequence** |
| MrUGT78W1 S147A R1 | GAGCGAGCATGGAAATGCCACCCACACCGGG |
| MrUGT78W1 S147A F1 | CCCGGTGTGGGTGGCATTTCCATGCTCGCTC |
| MrUGT78W1 T349A R1 | GCGAGTACTTGGGTCTGATTTGCCCATGGCACTATT |
| MrUGT78W1 T349A F1 | AATAGTGCCATGGGCAAATCAGACCCAAGTACTCGC |
| MrUGT78W1 N350P R1 | GCGAGTACTTGGGTCTGAGGTGTCCATGGCACTATT |
| MrUGT78W1 N350P F1 | AATAGTGCCATGGACACCTCAGACCCAAGTACTCGC |
| MrUGT78W1 T352V R1 | GCGAGTACTTGCACCTGATTTGTCCATGGCACTATT |
| MrUGT78W1 T352V F1 | AATAGTGCCATGGACAAATCAGGTGCAAGTACTCGC |
| MrUGT78W1 S359A R1 | GTGTCACAAAGACACCTATTGCAGTATGTGCGAGTA |
| MrUGT78W1 S359A F1 | TACTCGCACATACTGCAATAGGTGTCTTTGTGACAC |
| MrUGT78W1 N370G R1 | TACTCTCGTACACAGAGCCGCAACCACAGTGTGTCA |
| MrUGT78W1 N370G F1 | TGACACACTGTGGTTGCGGCTCTGTGTACGAGAGTA |
| MrUGT78W1 N370S R1 | TACTCTCGTACACAGATGAGCAACCACAGTGTGTCA |
| MrUGT78W1 N370S F1 | TGACACACTGTGGTTGCTCATCTGTGTACGAGAGTA |
| MrUGT78W1 F387I R1 | ACCATCCGTCCAGTCATTCGTTGGTCACCAAAGATCGGCC |
| MrUGT78W1 F387I F1 | GGCCGATCTTTGGTGACCAACGAATGACTGGACGGATGGT |
| MrUGT78W1 F388L R1 | ACCATCCGTCCAGTCATTCGTTGGTCACCCAAGAACGGCC |
| MrUGT78W1 F388L F1 | GGCCGTTCTTGGGTGACCAACGAATGACTGGACGGATGGT |
| MrUGT78W1 D390A R1 | ACCATCCGTCCAGTCATTCGTTGGGCACCAAAGAACGGCC |
| MrUGT78W1 D390A F1 | GGCCGTTCTTTGGTGCCCAACGAATGACTGGACGGATGGT |
| MrUGT78W1 Q391N R1 | ACCATCCGTCCAGTCATTCGATTGTCACCAAAGAACGGCC |
| MrUGT78W1 Q391N F1 | GGCCGTTCTTTGGTGACAATCGAATGACTGGACGGATGGT |
| MrUGT78W1 Q391H R1 | ACCATCCGTCCAGTCATTCGGTGGTCACCAAAGAACGGCC |
| MrUGT78W1 Q391H F1 | GGCCGTTCTTTGGTGACCACCGAATGACTGGACGGATGGT |

**Table S4.** Primers for transient expression. Restriction sites are underlined.

| **Gene** | **Primer** | **Sequence** |
| --- | --- | --- |
| SK-MrUGT78R1 | Forward | cgctctagaactagtGGATCCATGGCCCAAAGCTCGCCGGA |
|  | Reverse | gataagcttgatatcGAATTCTTATTTGGAGATCACCTCTACCAAAC |
| SK-MrUGT78R2 | Forward | cgctctagaactagtGGATCCATGACACAAAGCTCGCAGGA |
|  | Reverse | gataagcttgatatcGAATTCTTATTTGGAAATTACCTCCACCAATGTAT |
| SK-MrUGT78W1 | Forward | cgctctagaactagtGGATCCATGGCTGGGACGGAAAGTTCTGAT |
|  | Reverse | gataagcttgatatcGAATTCTTAAGACGTAGATATTAAGTCCACCAG |
